# Four-Coordinate Co(III) Imide with an Unusually Tilted Terminal Imido Ligand

**DOI:** 10.1021/acs.organomet.3c00473

**Published:** 2024-01-12

**Authors:** Li Gu, Addison Fraker, Niklas B. Thompson, Alex McSkimming

**Affiliations:** † Department of Chemistry, Tulane University, New Orleans, Louisiana 70118, United States; ‡ Chemical Sciences and Engineering Division, Argonne National Laboratory, Lemont, Illinois 60439, United States

## Abstract

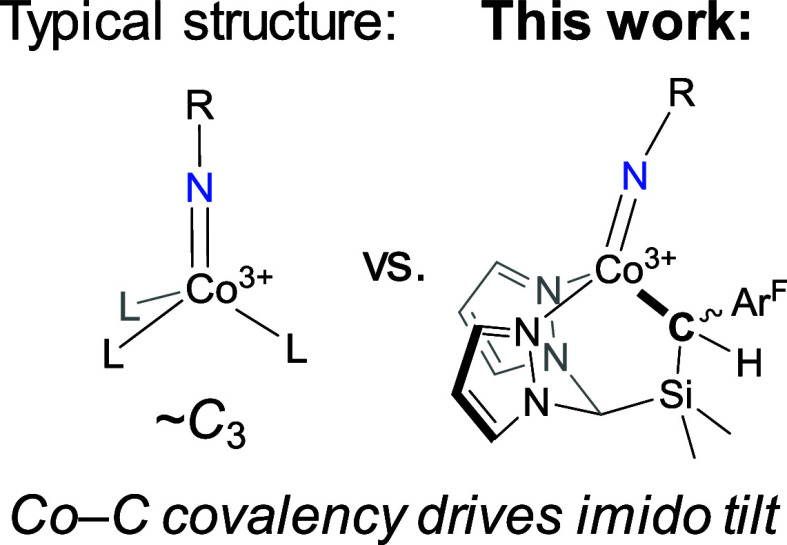

We report herein the synthesis and characterization of a terminal Co(III) imido complex supported by an intermediate field N,N,C heteroscorpionate. This chemistry is enabled through the development of an additional member of this ligand type featuring Ph_2_(CH_3_)C– substituents, one of which weakly binds and stabilizes Co in the corresponding Co(I) precursor. The Co(III) imide is low-spin with no evidence for thermal population of open-shell excited states. Unusually, the imido ligand in this molecule tilts markedly toward the C_alkyl_ donor. DFT calculations suggest this structural feature to be largely a result of strong Co–C covalency, underscoring the importance of M–C bonding in determining the (electronic) structure of metal centers supported by this class of ligand.

## Introduction

Late transition metal imido complexes (M=N_im_R) have long attracted the interest of the inorganic community, due in part to their unusual M–N_im_ multiple bonding^1−3^ and role as intermediates in nitrene transfer reactions.^4,5^ Terminal Co(III) imides form a particularly well-studied subset of these molecules.^6−23^ The connection between Co–N_im_ covalency and N_im_-centered reactivity is well established, with these properties being able to be dramatically altered through changes in the supporting ligand field. If bound by strong-field donors,^6,7,11,12,15,19,22,23^ and indeed some weak-field donors,^8,17^ Co imides feature diamagnetic ground states, highly covalent Co–N_im_ bonding, and remarkable thermal stabilities. When supported by relatively weak-field ligands, however, such imido complexes feature thermal population of open-shell excited states,^10,14,16^ or are strictly high-spin (*S* = 2),^14,18,21^ which results, for example, in dramatically increased rates of H atom abstraction at N_im_.^10,14,16,18,21^ These observations are exemplified, on the one hand, by four-coordinate Co imido complexes supported by tris(phosphino)borates, which are incredibly robust,^6^ and, on the other hand, those bound by tris(pyrazolyl)borates (Tp), which are considerably more reactive.^10^

**Scheme 1:**
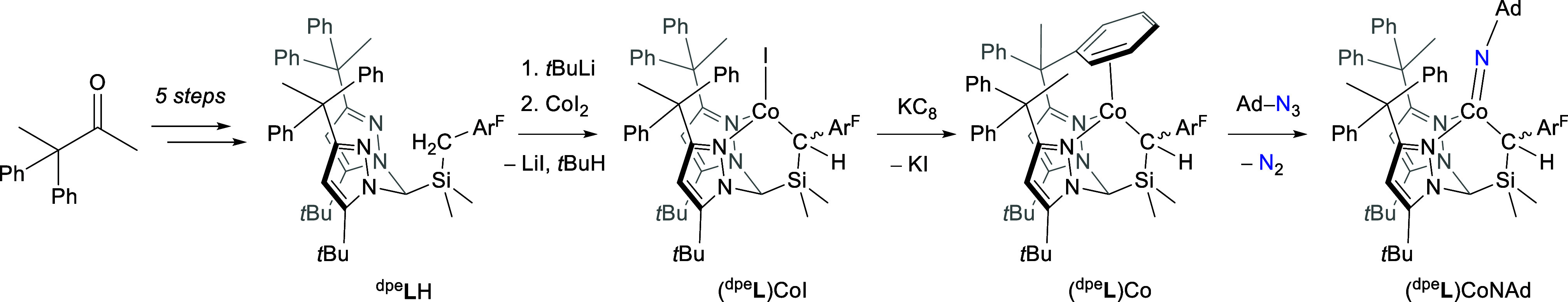
Synthesis of ^dpe^**L**H and Co Complexes of ^dpe^**L** Ar^F^ = 3,5-(CF_3_)C_6_H_3_, Ad = 1-adamantyl.

We have recently reported a new class of N,N,C “heteroscorpionate” ligands ^R^**L** (where ‘R’ refers to the metal-adjacent pyrazole substituents) containing a strongly basic alkyl donor and two weak-field pyrazoles (Scheme 1).^24,25^ Four-coordinate Fe(III) imides supported by these ligands are intermediate-spin (*S* = 3/2),^24^ in contrast to Fe(III) imides bound by strong-field scorpionates, which are invariably low-spin (*S* = 1/2),^7,26,27^ and those in weak fields, which are high-spin (*S* = 5/2).^28^ In light of this reported Fe chemistry, we were curious to probe the (electronic) structure of Co(III) imido complexes bound by ^R^**L** ligands. In particular, we were curious to ascertain the impact of moving away from the approximate *C*_3_ symmetric ligand environment shared by all reported trigonally coordinated Co imides.^6,7,9−12,15,19,22,23^ Herein, we report the new ^dpe^**L** (dpe = Ph_2_(CH_3_)C−) ligand, the corresponding Co(I) complex, and its transformation to a Co(III) imide. The latter species is unusually distorted away from pseudo 3-fold symmetry, with the imido ligand distinctly tilted toward the C_alkyl_ donor. DFT calculations suggest that this structural feature arises due to the highly covalent Co–C bond, further highlighting the pronounced impact of the M–C interaction on the structure and bonding of ^R^**L**-supported metal centers.

## Results and Discussion

**Figure 1:**
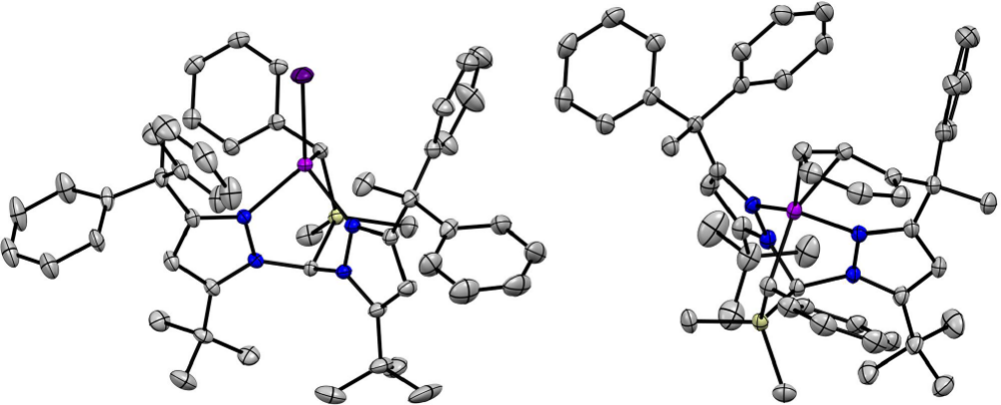
Thermal ellipsoid plots (50%) of (^dpe^**L**)CoI (left) and (^dpe^**L**)Co (right). Pink, purple, blue, yellow, and gray ellipsoids represent Co, I, N, Si, and C, respectively. Hydrogen atoms, solvent molecules, and CF_3_ groups are omitted for clarity.

We previously reported the synthesis of Co(I) complex [(^*t*Bu^**L**)CoN]_2_, which is a natural precursor for the synthesis of Co(III) imides. This species, however, is not only poorly thermally stable, but quite difficult to prepare from (^*t*Bu^**L**)CoI, as this reaction requires careful monitoring to prevent over-reduction and decomposition.^25^ Taking inspiration from the dipyrrin ligand systems of the Betley group,^16,17^ we sought to replace the metal-adjacent *t*Bu groups in ^*t*Bu^**L** with substituents capable of engaging in weak Co(I)-arene interaction(s). We predicted that such putative complexes would exhibit considerably improved robustness relative to [(^*t*Bu^**L**)CoN]_2_, thus serving as more tractable precursors to Co(III) imides. Simple modeling suggested trityl (Ph_3_C−) groups would be overly large and prevent metal coordination; the smaller Ph_2_(CH_3_)C– or ‘dpe’ group was therefore selected instead. Starting from Ph_2_(CH_3_)C(COCH_3_), the new ligand precursor ^dpe^**L**H was prepared analogously to our previously reported 6-*t*Bu-1,1-Me_2_-indan substituted analogue^25^ in 8% overall yield on a multigram scale (see Experimental Section). Likewise, deprotonation of ^dpe^**L**H using *t*BuLi followed by metalation with CoI_2_ gave the corresponding high-spin (*S* = 3/2) Co(II) complex (^dpe^**L**)CoI as a dark green crystalline solid in ∼60% yield (Scheme 1 and Figure 1). Reduction of (^dpe^**L**)CoI proved straightforward: most conveniently, stirring Et_2_O solutions of (^dpe^**L**)CoI over an excess of KC_8_ gave the dark red-orange, Co(I) complex (^dpe^**L**)Co (Scheme 1 and Figure 1) in good yield (∼80%). In contrast to the reduction of (^*t*Bu^**L**)CoI, prolonged reaction times for (^dpe^**L**)CoI led to very little degradation, allowing for the isolation of bulk samples of (^dpe^**L**)Co in highly reproducible yields and purity. In addition, (^dpe^**L**)Co exhibits markedly improved thermal stability compared to [(^*t*Bu^**L**)CoN]_2_: heating a C_6_H_6_ solution of the latter at 50 °C results in near complete decomposition in an hour, whereas (^dpe^**L**)Co decomposes relatively slowly under these conditions (Figure S24).

As expected, structural data reveal the reduction of (^dpe^**L**)CoI to (^dpe^**L**)Co to result in displacement of iodide by a Ph group of the Ph_2_C(CH_3_)– substituent, which binds the metal in an η^2^ fashion (Figure 1). We have observed that M–C_alkyl_ distances for this class of heteroscorpionates are essentially static across oxidation states.^24,25^ Surprisingly, then, the Co–C_alkyl_ bonds for (^dpe^**L**)Co are appreciably lengthened compared to (^dpe^**L**)CoI: 2.127(3) vs 2.064(2) Å, respectively. This is accompanied by a slight contraction of the Co–N_pz_ distances (∼0.03 Å). These changes are potentially due to the steric pressure imparted by the η^2^–Ph ligand closely abutting the Ar^F^ group, which may push the Co and C_alkyl_ centers apart. Consistently, ^19^F NMR spectra for (^dpe^**L**)Co show two broad resonances for the Ar^F^ CF_3_ groups, suggesting that rotation about the C_alkyl_–Ar^F^ bond is relatively slow c.f. (^dpe^**L**)CoI, for which only one ^19^F signal is observed. The Co-coordinated C–C π-bond is elongated somewhat to 1.405(4) Å, with the remainder of the bond lengths within the arene being likewise consistent with a slight disruption of aromaticity. Paramagnetically shifted NMR resonances and a solution-state magnetic moment of 3.4 μ_B_ for (^dpe^**L**)Co confirm an *S* = 1 state at Co with a large orbital contribution; observed moments of >3.2 μ_B_ are not uncommon for four-coordinate, high-spin Co(I) complexes.^25,29,30^

**Figure 2:**
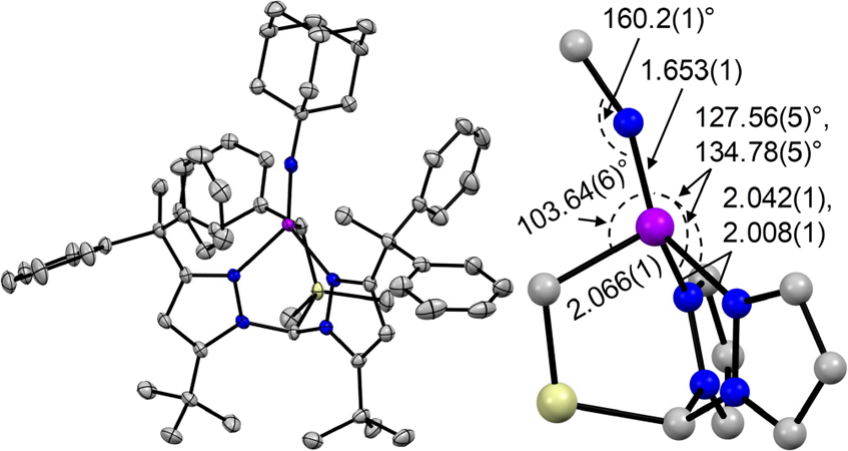
Thermal ellipsoid plot (50%) for (^dpe^**L**)CoNAd (left) and a truncated model showing selected bond lengths and angles (right). All bond distances are given in Å. The color scheme is the same as that for Figure 1. Hydrogen atoms, solvent molecules, and CF_3_ groups are omitted for clarity.

**Figure 3:**
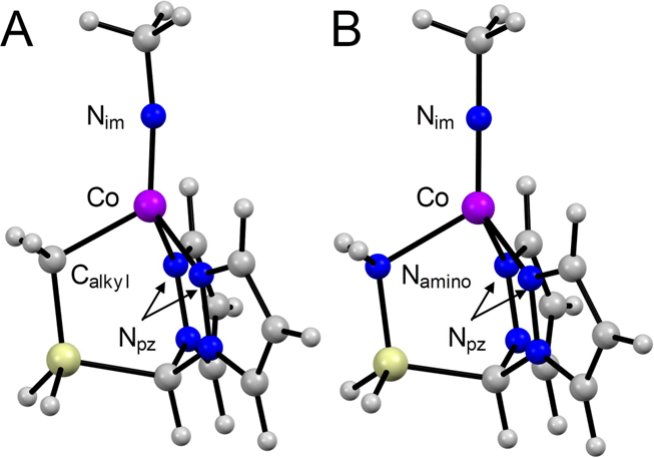
Labeling schemes for the truncated structures (A) (*L)CoNCH_3_ and (B) [(**L)CoNCH_3_]^+^.

Reaction of (^dpe^**L**)Co with 1-adamantyl azide rapidly liberated N_2_, cleanly giving the corresponding Co(III) imido complex (^dpe^**L**)CoNAd (Figure 2) as a green-brown crystalline solid. As per most other Co(III) imides,^6−8,11,12,15,17,19,22,23^ (^dpe^**L**)CoNAd is closed shell (*S* = 0) as indicated by its NMR spectra and solution magnetometry, with negligible thermal population of excited states at room temperature (RT). In line with this, (^dpe^**L**)CoNAd is quite thermally stable, decomposing only slowly in toluene at 50 °C to a complex mixture of diamagnetic products (∼60% degraded in 24h). The Co–ligand bond lengths are all within usual ranges; for example, the Co–N_im_ distance of 1.653(1) Å is typical.^10−12,19^ A notable feature in the solid state structure of (^dpe^**L**)CoNAd is a pronounced tilt of the imido ligand toward the C_alkyl_ donor (∠C_alkyl_–Co–N_im_ = 103.64(6)°), resulting in quasi-planarization of the N_im_–Co–N_pz_–N_pz_ atoms with Co approaching a trigonal pyramidal geometry (τ_4_ = 0.70). We initially attributed this structural distortion to the large steric profile of the proximal Ad and Ph_2_C(CH_3_)– groups, as all other reported four-coordinate Co(III) N–alkyl imidos are pseudo *C*_3_; i.e., with all L_donor_–Co–N_im_ angles being roughly equal.^10−12,19^ Close inspection of the space-filling model for (^dpe^**L**)CoNAd (Figure S25), however, suggested that the Ad group is not sufficiently well-ensconced within the ^dpe^**L** ligand to induce the observed structure. Furthermore, DFT calculations performed on a highly truncated model of (^dpe^**L**)CoNAd (i.e., (*L)CoNCH_3_; Figure 3) reproduce the approximate trend in angles about Co observed for (^dpe^**L**)CoNAd; e.g., ∠Co–N_im_–CH_3_ = 149° and ∠C_alkyl_–Co–N_im_ = 96°, whereas TpCoNCH_3_ is predicted to be rigorously *C*_3v_ (Figure S26). Replacing Si with C furnishes a similarly skewed structure (Figure S27), suggesting that any changes in Si–C(σ) → Co(3*d*) bonding has little impact (in stark contrast to (^*t*Bu^**L**)Co vs [(^*t*Bu^**L**)CoN]_2_).^25^

**Figure 4:**
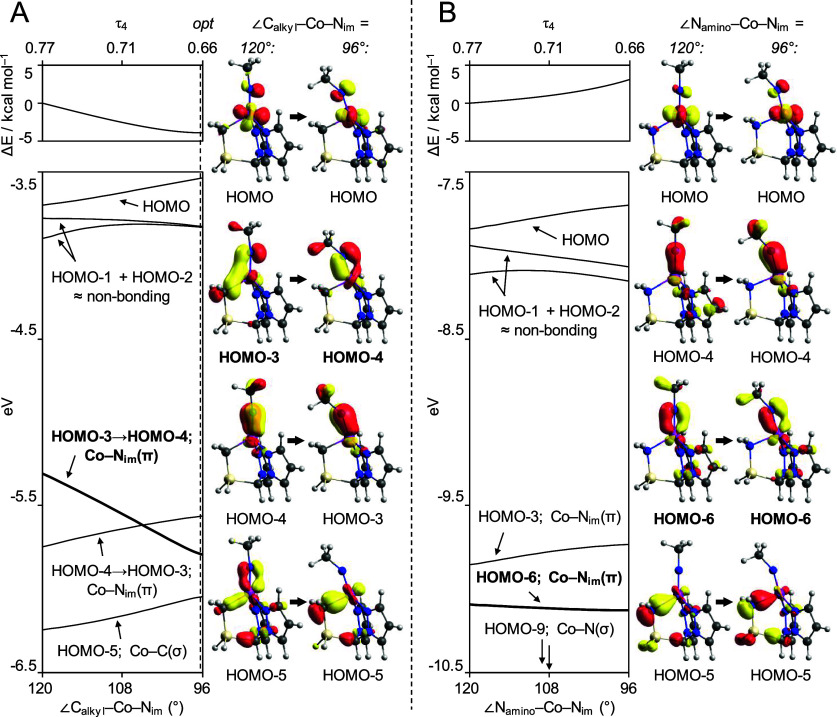
Calculated adiabatic potential energy surfaces as (A) ∠C_alkyl_–Co–N_im_ or (B) ∠N_amino_–Co–N_im_ moves from 120° to 96°, the corresponding Walsh diagrams and selected MOs for: (A) (*L)CoNCH_3_ and (B) (*L)CoNCH_3_ upon making the substitution CH_2_ → NH_2_^+^ to give [(**L)CoNCH_3_]^+^. For (B) the HOMO–4 and HOMO–5 have minimal Co(3*d*) contributions and so are omitted for clarity. Key Co–N_im_(π) MOs are highlighted. Isosurfaces are shown at 0.07 au.

Intrigued by the observations presented above, we calculated the structures of (*L)CoNCH_3_ scanning ∠C_alkyl_–Co–N_im_ from 120° to the computed optimum value of 96°. The adiabatic potential energy surface (APES) is relatively flat over this range, with the electronic energy decreasing by roughly 4 kcal mol^–1^ overall (Figure 4A). While the Co–N_im_ distance remains largely unchanged, the Co–C_alkyl_ bond shortened appreciably from 2.04 to 2.00 Å. This occurs together with a less pronounced increase in the Co–N_pz_ distances of ∼0.01 Å (Figure S28). The Co–N_im_–CH_3_ angle changes markedly from 173 to 149°. The associated Walsh diagram (Figure 4A) is instructive: the HOMO, HOMO–1, and HOMO–2 are approximately nonbonding Co 3*d* orbitals, with the HOMO–1 and HOMO–2 remaining substantially unchanged throughout. The HOMO has partial Co–N_im_ π* character and becomes increasingly repulsive as the imido ligand tilts toward the C_alkyl_ donor. This is mitigated somewhat by rehybridization at N_im_, which is reflected by the bending of the Co–N_im_–CH_3_ group and the increasing N_im_(2s) character (0.5% → 4.2%) in the corresponding localized Co–N_im_(π) MO. Constraining the ∠Co–N_im_–CH_3_ angle to be 180° and scanning the C_alkyl_–Co–N_im_ angle qualitatively reproduces the changes observed for the unrestricted calculation (Figure S29). Here, however, the ideal tilt for the imido is much less distinct, with ∠C_alkyl_–Co–N_im_ ≈ 112°. As anticipated, then, preventing rehybridization at N_im_ causes the HOMO to increase in energy far more rapidly, which manifestly disfavors substantial bending of the C_alkyl_–Co–N_im_ moiety.

By far, the largest change in orbital energy for (*L)CoNCH_3_ occurs for the HOMO–3, which is one of the two primary Co–N_im_(π) bonding MOs. At ∠C_alkyl_–Co–N_im_ = 120°, the Co(3*d*)–N_im_(2*p*) interaction is heavily mixed with the C_alkyl_(2*p*) orbitals. As this angle decreases, however, Co(3*d*)–N_im_(2*p*) overlap improves markedly with the C_alkyl_(2*p*) contributions simultaneously diminished. As C_alkyl_(2*p*)-Co(d) overlap for the HOMO–3 is relatively poor throughout, the increase in Co–N_im_(π) bonding is more than sufficient to compensate for the loss of Co–C_alkyl_(σ) character; this results in the energy of this MO dropping precipitously. Concomitantly, the HOMO–5 loses almost all Co–N_im_(π) character and is destabilized, albeit to a much lesser extent, transforming into a highly localized Co–C_alkyl_(σ) MO. The HOMO–4 consists of the orthogonal Co–N_im_(π) bond, which has a small Co–N_pz_(σ*) component. Tilting the imido ligand marginally increases the σ* character (thus lengthening the Co–N_pz_ bonds); this raises the HOMO–4 in energy until it exchanges positions with the HOMO–3 in the orbital manifold at ∠C_alkyl_–Co–N_im_ ≈ 105°. The combined destabilization of the HOMO, HOMO–4, and HOMO–5 is clearly insufficient to offset stabilization of the HOMO–3, hence tilting of the imido ligand in (*L)CoNCH_3_ is favored.

Making the isoelectronic and isolobal substitution CH_2_ → NH_2_^+^ for (*L)CoNCH_3_ (giving [(**L)CoNCH_3_]^+^, Figure 3) and scanning ∠N_amino_–Co–N_im_ from 120° to 96° is revealing. For this species, tilting the imido toward the (now) N_amino_ donor is in fact disfavored by ∼4 kcal mol^–1^, despite the trends in Co–ligand bond lengths being very similar to those observed for the parent (*L)CoNCH_3_ (Figure S28). The associated Walsh diagram is shown in Figure 4B, and makes the reason for this apparent. The HOMO–6 consists of an analogous Co–N_im_(π) orbital to the HOMO–3 in the parent (*L)CoNCH_3_. In contrast to (*L)CoNCH_3_, however, the considerably less covalent Co–N_amino_ interaction c.f. Co–C_alkyl_ introduces only minor N_amino_(2*p*) character into this orbital. Consequently, contracting ∠N_amino_–Co–N_im_ does little to affect Co–N_im_(π) overlap, and so the energy of this MO lowers only slightly. This is evidently insufficient to counteract increasing repulsion in the HOMO and in the orthogonal Co–N_im_(π) MO as the imido tilts toward the amino donor.

Applying quantum theory of atoms in molecules (QTAIM) analysis to (*L)CoNCH_3_ and [(**L)CoNCH_3_]^+^ provides additional insight. The delocalization index (DI), a measure of bond order,^31^ for the Co–C_alkyl_ and Co–N_amino_ interactions both increase slightly upon tilting the imido (0.787 → 0.824; Δ0.037 and 0.517 → 0.588; Δ0.071, respectively). At the same time, a comparable decrease in Co–N_im_ DIs is observed: 1.909 → 1.867; Δ0.042 and 2.089 → 2.015; Δ0.074, respectively. One possible interpretation of this is that for (*L)CoNCH_3_, the imido group tilts in order to increase the covalency of the Co–C_alkyl_ interaction, which occurs at the expense of Co–N_im_ bonding. In contrast, the Co–N_amino_ bond for [(**L)CoNCH_3_]^+^ is insufficiently covalent (ΔDI ≈ –0.25 c.f. Co–C_alkyl_ in (*L)CoNCH_3_) to overcome the weakening of the Co–N_im_ interaction that such a distortion entails.

## Conclusions

We have shown that incorporation of a metal-adjacent Ph_2_(CH_3_)C− group into a supporting ligand, herein the N,N,C heteroscorpionate ^dpe^**L**, to be a useful method for the stabilization of otherwise highly thermally sensitive Co(I) centers. This strategy is currently being adapted toward the isolation of other reactive, low-valent first-row transition metal complexes. The four-coordinate terminal Co(III) imide supported by ^dpe^**L** and reported herein features an imido ligand that tilts markedly toward the C_alkyl_ donor. We attribute this structural anomaly to the highly covalent Co–C_alkyl_ bond, which drives a noncanonical rehybridization at the imido N atom. Others have reported pronounced increases in N_im_-centered reactivity for Co(III) imides that are forced, through sterics, from attaining their preferred geometry.^17^ We hypothesize that similar principles may be applied to rationally tune the reactivity of imido complexes bound by our (and other) heteroscorpionate ligands; e.g., by increasing bulk around the C_alkyl_ donor and decreasing bulk on the pyrazoles.

## Experimental Section

### General Methods

All manipulations involving metal complexes were carried out in a N_2_ atmosphere glovebox. Glassware was oven-dried for at least several hours at 160 °C prior to use.

### Materials

All solvents except *n*-pentane were distilled from purple Na/benzophenone prior to use. All solvents were stored over activated 3 Å molecular sieves for at least 24 h prior to use. All reagents were purchased from commercial suppliers and used without further purification, unless otherwise noted. 3,3-diphenyl-2-butanone^32^ was prepared according to literature procedures.

### Comment on Purity

Organic products were deemed pure by ^1^H NMR spectroscopy. All metal complexes were isolated as crystalline solids that were homogeneous under microscope inspection. The purity of metal complexes was readily assessed by a combination of ^1^H and ^19^F NMR spectroscopies. The latter proved particularly useful, as the low symmetry and paramagnetism of (^dpe^**L**)CoI and (^dpe^**L**)Co rendered thorough interpretation of ^1^H NMR spectra difficult. The total integrated area for impurity peaks constituted <3% of the ^19^F content for all reported complexes. All metal complexes formed homogeneous solutions in noncoordinating solvents, e.g., C_6_H_6_, thus precluding the presence of inorganic salts. All reported spectra are provided in the Supporting Information.

### Spectroscopy and Spectrometry

NMR spectra were recorded on a Bruker 300 MHz spectrometer. ^1^H and ^13^C chemical shifts are reported in parts per million (ppm) relative to tetramethylsilane using residual solvent as an internal standard. ^19^F chemical shifts are reported in ppm relative to 5% v/v internal PhF.^33^ Solution-phase effective magnetic moments were determined by the method described by Evans^34^ and are corrected for diamagnetic contributions.^35^ Mass spectrometry data were collected on a Bruker micrOTOF II instrument with an ESI source. FTIR spectra were recorded on solid samples using a Bruker Alpha II FTIR spectrometer operating at 4 cm^–1^ resolution.

### X-ray Crystallography

Low-temperature diffraction data were collected on a Bruker-AXS X8 Kappa Duo diffractometer coupled to an APEX2 CCD detector. The data collections were executed with Mo *K*_α_ radiation (λ = 0.71073 Å) from a *I*μ*S* microsource, performing ϕ-and ω-scans. Absorption and other corrections were applied using the program SADABS.^36,37^ The structures were solved by dual-space methods using SHELXT^38^ and refined against *F*^2^ on all data by full-matrix least-squares with SHELXL-2017,^39^ following established refinement strategies.^40^ All non-hydrogen atoms were refined anisotropically, and all hydrogen atoms were included into the model at geometrically calculated positions and refined using a riding model.

### Computational Details

All calculations were carried out using revision 5.0.2 of the ORCA suite of programs.^41^ DFT calculations made use of the “TIGHTSCF” convergence criteria; unless stated otherwise, default settings were used for all other methods.

Truncated models of ^dpe^**L** were constructed by replacing the *t*Bu pyrazole substituents, Ar^F^ group, and Si-bound CH_3_ groups with H (*L). This was anticipated to qualitatively model the donor properties of *L, while reducing computational cost and easing interpretation of calculated MOs. **L was constructed by replacing the C_alkyl_ donor atom in *L with an N atom. All atoms were allowed to relax along the ground-state potential energy surface using the BP86 exchange-correlation functional with the def2-TZVP basis set.^42−44^ The contracted auxiliary Coulomb fitting basis def2/*J* was applied to all atoms.^45^ Grimme’s atom-pairwise correction with Becke-Johnson damping (D3BJ) was included to account for the effects of dispersion.^46^ Localized MOs were constructed using the intrinsic bond orbital (IBO) method developed by Knizia.^47^ QTAIM analysis was performed using AIMAll^48^ using electron densities calculated in ORCA.

### Synthesis of 5-*t*Bu-3-Ph_2_(CH_3_)C-pyrazole

To a suspension of NaH (9.6 g, 0.41 mol) in THF (600 mL; predrying not required), was cautiously added a solution of 3,3-diphenyl-2-butanone (30.0 g, 0.134 mol) and methyl trimethyl acetate (32.0 g, 0.275 mol) in THF (50 mL). The resulting suspension was stirred at 65 °C for 48 h, at which point a dark orange-brown solution had formed with some suspended NaH still visible. The mixture was cooled to RT, and water (∼100 mL) was added cautiously to quench the remaining NaH. The mixture was made strongly acidic with HCl (6 M, ∼200 mL) and diluted with hexanes (200 mL). The organic layer was separated, dried over Na_2_SO_4_, and volatiles were removed under reduced pressure. The resulting brown oil was redissolved in EtOH (200 mL) and hydrazine monohydrate (25 mL, ∼0.5 mmol) added. The mixture was heated to 80 °C for 3 h, at which point the now yellow solution was cooled and water (∼1000 mL) was added slowly to induce precipitation of the impure product as a pale-yellow solid. This was collected by filtration, washed with copious water, and sucked dry. The crude solid was dissolved in the minimum of CH_2_Cl_2_, diluted with 3 volumes of MeCN, and concentrated until the CH_2_Cl_2_ had fully evaporated, leaving a pale-yellow crystalline solid and a dark yellow supernatant. The mixture was cooled to RT, and the product was allowed to completely precipitate. Solids were then collected by filtration and washed with MeCN (3 × 30 mL). Yield: 24.8 g (61% over 2 steps). ^1^H NMR (300 MHz, CDCl_3_) δ 7.35–7.15 (m, 10H, Ar*H*), 5.89 (s, 1H, pz*H*), 2.13 (s, 3H, Ph_2_C*H*_3_C), 1.32 (s, 9H, C(C*H*_3_)_3_); the NH proton could be observed as a broad peak at δ 10, although this value varied. ^13^C{^1^H} NMR (101 MHz, CDCl_3_) δ 157.22 (Ar*C*), 154.30 (Ar*C*), 148.02 (Ar*C*), 128.05 (Ar*C*), 128.04 (Ar*C*), 126.31 (Ar*C*), 101.22 (Ar*C*), 48.50 (Ph_2_CH_3_*C*), 31.53 ((CH_3_)_3_*C*), 30.46 ((*C*H_3_)_3_C), 29.33 (CPh_2_*C*H_3_). ESI-MS (+): *m*/*z* 305.1984; calc. for [M + H]^+^: *m*/*z* 305.2017.

### Synthesis of (5-*t*Bu-3-Ph_2_(CH_3_)C-pyrazole)_2_CH_2_

To the solution of 5-*t*Bu-3-Ph_2_(CH_3_)C-pyrazole (16.0 g, 0.0526 mol) and BzNEt_3_Cl (1.1 g, 4.8 mmol) in CH_2_Cl_2_ (250 mL) and CH_2_Br_2_ (50 mL) was added a freshly made, RT solution of NaOH (400 mL, aq. 50% w/w). The biphasic mixture was stirred rapidly at RT overnight. The mixture was then transferred to a separatory funnel, and the lower aqueous layer was discarded. The organic layer was diluted with hexanes (400 mL) and washed with additional water (∼500 mL). The organic layer was separated, dried over Na_2_SO_4_, and volatiles were removed under reduced pressure to afford a crude oily solid that consisted of a regioisomeric mixture of CH_2_–bridged pyrazoles. This was dissolved completely in the minimum CH_2_Cl_2_ (∼20 mL) and diluted with 3 volumes of hexane. The solution was then boiled vigorously on a hot plate until all the CH_2_Cl_2_ had evaporated. This was then cooled to RT, resulting in the deposition of the product in a near-pure state as a colorless crystalline solid. This was collected by filtration and washed with a portion of hexanes (∼10 mL). The crude product was dissolved again in the minimum CH_2_Cl_2_, diluted with 3 volumes of MeCN, and the solution concentrated until the CH_2_Cl_2_ had completely evaporated. Slowly cooling the solution to RT resulted in crystallization of the product, which was collected by filtration and washed with MeCN (3 × 5 mL). Yield: 3.20 g (20%). ^1^H NMR (300 MHz, CDCl_3_) δ 7.24–7.11 (m, 20H, 20 × Ar*H*), 6.44 (s, 2H, 2 × pz*H*), 5.73 (s, 2H, pz_2_–C*H*_2_), 2.04 (s, 6H, 2 × Ph_2_C*H*_3_C), 1.23 (s, 18H, 2 × C(C*H*_3_)_3_). ^13^C{^1^H} NMR (101 MHz, CDCl_3_) δ 156.66 (Ar*C*), 152.96 (Ar*C*), 149.08 (Ar*C*), 128.36 (Ar*C*), 127.60 (Ar*C*), 125.69 (Ar*C*), 104.71 (Ar*C*), 64.93 (pz_2_-*C*H_2_), 49.22 (Ph_2_CH_3_*C*), 31.70 ((CH_3_)_3_*C*), 30.34 ((*C*H_3_)_3_C), 29.20 (Ph_2_*C*H_3_C). ESI-MS (+): *m*/*z* 621.3935; calc. for [M + H]^+^: *m*/*z* 621.3957.

### Synthesis of ^dpe^**L**H

A solution of (5-*t*Bu-3-Ph_2_(CH_3_)C-pyrazole)_2_CH_2_ (2.10 g, 3.38 mmol) in THF (25 mL) was cooled to −78 °C in the glovebox cold well and a solution of *n*BuLi in hexanes (2.5 M, 1.5 mL, 3.7 mmol) was added dropwise *via* syringe. This resulting yellow solution was stirred at −78 °C for 6 h, at which point it was added slowly *via* pipet to a precooled, stirred solution of Me_2_SiCl_2_ (1.4 mL, 12 mmol) in THF (4 mL) at −78 °C. The pale orange reaction mixture was allowed to come slowly to RT, stirred for 30 min, and volatiles were removed thoroughly under reduced pressure to yield a white solid. The crude chlorosilane was sufficiently pure for the next step and did not require separation from LiCl. The crude solid was dissolved/suspended in Et_2_O (∼15 mL), and an Et_2_O (∼20 mL) solution of (3,5-(CF_3_)_2_C_6_H_3_)CH_2_MgCl was added slowly *via* pipet at RT. The Grignard reagent was prepared from (3,5-(CF_3_)_2_C_6_H_3_)CH_2_Cl (1.34 g, 5.10 mmol) according to our previously reported procedure.^24^ After 2h of stirring at RT, the reaction mixture was removed from the glovebox and quenched by dropwise addition of water. The organic layer was separated, dried over Na_2_SO_4_, and solvent removed under reduced pressure to yield a yellow oil. This was dissolved in a small amount of CH_2_Cl_2_ (∼1 mL) and diluted with MeOH (∼10 mL). The solution was then boiled vigorously on a hot plate until all the CH_2_Cl_2_ had evaporated, resulting in deposition of a very pale-yellow oil. The mixture was placed in an −10 °C freezer for several hours, causing the oil to solidify. The MeOH supernatant was decanted, and the crystallization procedure was repeated once more. The resulting colorless solid was dried under high vacuum overnight to completely remove traces of MeOH. Yield: 2.10 g (69% over 2 steps). ^1^H NMR (300 MHz, (CD_3_)_2_CO) δ 7.67 (s, 1H, *p*-Ar^F^*H*), 7.45 (s, 2H, 2 × *m*-Ar^F^*H*), 7.27–7.12 (m, 20H, 20 × Ar*H*), 6.77 (s, 1H, pz_2_–C*H*), 6.00 (s, 2H, 2 × pz*H*), 2.33 (s, 2H, C*H*_2_-Ar^F^), 2.12 (s, 6H, 2 × Ph_2_C*H*_3_C), 1.12 (s, 18H, 2 × C(C*H*_3_)_3_), 0.00 (s, 6H, Si(C*H*_3_)_2_). ^13^C{^1^H} NMR (101 MHz, CDCl_3_) δ 155.91 (Ar*C*), 153.67 (Ar*C*), 148.81 (Ar*C*), 148.73 (Ar*C*), 143.14 (Ar*C*), 131.09 (q, *J*_CF_ = 30 Hz, *m*-Ar^F^*C*), 128.42 (Ar*C*), 128.35 (Ar*C*), 128.25 (m, *o*-Ar^F^*C*), 127.64 (Ar*C*), 125.85 (Ar*C*), 125.83 (Ar*C*), 123.61 (q, *J*_CF_ = 270 Hz, *C*F_3_), 117.94 (sept, *J*_CF_ = 4 Hz, *p*-Ar^F^*C*), 106.14 (Ar*C*), 73.50 (pz_2_-*C*H), 49.14 (Ph_2_CH_3_*C*), 32.25 (Ph_2_*C*H_3_C), 30.53 ((*C*H_3_)_3_C), 29.39 (*C*H_2_–Ar^F^), 25.45 ((CH_3_)_3_*C*), –1.97 (Si(*C*H_3_)_2_). Restricted rotation about the Ph_2_*C*H_3_C–pz bond renders the −Ph groups inequivalent, giving rise to 8 Ar*C* signals (one is obscured due to accidental equivalence). ^19^F NMR (282 MHz, (CD_3_)_2_CO): δ −62.67 (s, C*F*_3_). FTIR: cm^–1^ 3055w, 3026w, 2972m, 2868w, 1597m, 1529m, 1490m, 1462m, 1443w, 1368s, 13372, 1309w, 1274s, 1249m, 1209m, 1167s, 1129s, 1062m, 1025m, 990m, 918m, 890m, 845m, 812m, 759m, 748m, 729m, 696s, 680m, 666m, 647m, 610w, 566w, 537w, 507w, 458w. ESI-MS (+): *m*/*z* 905.4392; calc. for [^dpe^**L**H+H]^+^: *m*/*z* 905.4287

### Synthesis of (^dpe^**L**)CoI

A solution of ^dpe^**L**H (2.00 g, 2.21 mmol) in THF (∼25 mL) was cooled to −78 °C in the glovebox cold well, at which point a solution of *t*BuLi (0.90 mL, 2.7 M in heptane, 2.4 mmol) was added dropwise with stirring. Upon completion of this addition, the solution was transferred to the glovebox freezer, where it was maintained at −30 °C for 2 h (stirring here is not required). This step ensures complete deprotonation of ^dpe^**L**H. The cold solution was removed from the freezer, and solid CoI_2_ (1.03 g, 3.30 mmol) was added quickly with stirring. The initial suspension rapidly clarified to give an intense, dark green solution. The mixture was allowed to come to RT and stirred for another 30 min. THF was removed thoroughly under reduced pressure to give a dark green, oily solid. This was dissolved/suspended in C_6_H_6_ (∼30 mL), and all volatiles again were removed under reduced pressure. To the solid green residue was added C_6_H_6_ (∼50 mL), the mixture stirred briefly, and then one volume of pentane was added. This suspension was eluted through a short pad of SiO_2_ (∼4 cm) in a 50 mL glass frit, eluting with 1:1 benzene-pentane until the eluent was colorless. Volatiles were removed under reduced pressure to afford a crystalline green solid. The crude solid was suspended in Et_2_O (∼5 mL), and the suspension was stirred for 10 min before pentane (∼20 mL) was added to complete crystallization. The resulting green, crystalline solid was collected by filtration and washed with pentane (3 × 5 mL). Crystals suitable for XRD studies were obtained by storing a saturated Et_2_O solution of the complex at −30 °C for several days. Yield: 1.51 g (63%). RT magnetic moment (by the Evans method in C_6_D_6_): 4.4 μ_B_. ^1^H NMR (300 MHz, C_6_D_6_) δ 61.17 (1H), 60.13 (1H), 50.80 (1H), 21.40 (2H), 18.03 (3H), 15.28 (2H), 14.09 (2H), 13.50 (2H), 12.92 (1H), 11.93 (2H), 10.98 (1H), 3.54 (1H), 2.26 (1H), 2.11 (2H), 1.22 (3H), 0.87 (3H), 0.64 (2H), –0.44 (9H, C(C*H*_3_)_3_), –1.02 (9H, C(C*H*_3_)_3_), –5.29 (2H), –6.45 (4H), –11.50 (2H), −69.46 (1H). Signals for all 57 protons are observed. ^19^F NMR (282 MHz, C_6_D_6_): δ –100.01 (s, C*F*_3_). FTIR: cm^–1^ 3055w, 3026w, 2972m, 2936w, 2871w, 1597m, 1519m, 1491m, 1444m, 1410w, 1365s, 1291s, 1272m, 1235m, 1162s, 1123s, 1051w, 1026w, 996w, 944w, 884m, 835m, 749m, 696s, 681m, 665w, 624w, 542w, 494w, 442w. UV–vis (C_6_H_6_): λ_max_ (nm) ε_max_ (M^–1^ cm^–1^) 450 sh, 603 (430), 674 (620), 719 (820), 952 (110).

### Synthesis of (^dpe^**L**)Co

To a solution of (^dpe^**L**)CoI (200 mg, 0.183 mmol) in Et_2_O (∼5 mL) was added excess KC_8_ (40 mg, 0.30 mmol). The suspension was stirred at RT for 1 h, at which point the green mixture had changed to dark orange-red. The reaction was checked for completion using ^19^F NMR spectroscopy; if incomplete, stirring was continued for an additional hour. The mixture was filtered through a short pad of Celite, and volatiles were removed under reduced pressure. Pentane (∼2 mL) was added to the crude oily solid, and the resulting suspension/solution was stored at −30 °C overnight. The product crystallized as red blocks that were collected by filtration and washed with cold pentane (3 × 1 mL). Crystals suitable for XRD studies were obtained by storing a saturated tetramethylsilane solution of the complex at −30 °C overnight. Yield: 147 mg (81%). RT magnetic moment (by Evans method in C_6_D_6_): 3.4 μ_B_. ^1^H NMR (300 MHz, C_6_D_6_) δ 57.79 (1H), 47.37 (1H), 34.20 (1H), 21.31 (1H), 11.47 (1H), 10.65 (2H), 8.18 (1H), 6.51 (2H), 5.54 (1H), 4.47 (9H, C(C*H*_3_)_3_), 3.65 (3H), 2.79 (9H, C(C*H*_3_)_3_), 1.21 (4H), 0.86 (3H), –3.09 (3H), –5.51 (3H), –7.47 (2H), –7.68 (2H), –37.14 (1H), –39.01 (1H), –60.94 (1H). Signals for 4 protons were not observed. ^19^F NMR (282 MHz, C_6_D_6_): δ −82.79 (s, C*F*_3_), −84.33 (s, C*F*_3_). FTIR: cm^–1^ 3054w, 2970m, 2869w, 1590m, 1533m, 1490m, 1457m, 1443w, 1411w, 1363s, 1289w, 1270s, 1238m, 1197w, 1160s, 1116s, 1075w, 1049m, 1026m, 991m, 947m, 875m, 848m, 824m, 806m, 757m, 698m, 680m, 665m, 647w, 627w, 601w, 564w, 542w, 494w, 468w, 432w. UV–vis (C_6_H_6_): λ_max_ (nm) ε_max_ (M^–1^ cm^–1^) 385 (9.2 × 10^3^), 550 sh, (1.90 × 10^4^), 829 (670).

### Synthesis of (^dpe^**L**)CoNAd

AdN_3_ (9.2 mg, 0.052 mmol) in Et_2_O (∼1 mL) was added to a precooled suspension of (^dpe^**L**)Co (50 mg, 0.052 mmol) in Et_2_O (∼2 mL) at −78 °C in the glovebox cold well. The mixture was removed from the cold well, allowed to come to RT, and then filtered through a short pad of Celite (∼1 cm) in a glass pipet. Volatiles were removed under reduced pressure, and pentane (∼1 mL) was added to the crude residue. The resulting solution was placed at −30 °C for several days to yield dark brown crystals of the complex as a pentane solvate. These were collected by filtration and washed with cold pentane (3 × 1 mL). These crystals were suitable for XRD studies. Yield: 35 mg (61%). ^1^H NMR (300 MHz, C_6_D_6_) δ 7.79 (br, 2H, *o*-Ar^F^*H*), 7.54–7.50 (5H, m, 4 × Ar*H* + *p*-Ar^F^*H*), 7.44–7.37 (4H, m, 4 × Ar*H*), 7.28–7.17 (6H, m, 6 × Ar*H*), 7.14–7.04 (6H, m, 6 × Ar*H*), 6.04 (s, 1H, pz_2_–C*H*), 5.78 (s, 1H, pz*H*), 5.70 (s, 1H, pz*H*), 4.67 (s, 1H, C*H*–Ar^F^), 2.96 (3H, Ph_2_C*H*_3_C), 2.76 (3H, Ph_2_C*H*_3_C), 1.52 (d, *J* = 12 Hz, 3H, 3 × Ad*H*), 1.42 (d, *J* = 12 Hz, 3H, 3 × Ad*H*), ∼1.25 (3H, 3 × Ad*H*; partially concealed by (CH_3_)_2_(C*H*_2_)_3_), 1.10 (d, *J* = 12 Hz, 3H, 3 × Ad*H*), 0.99 (s, 9H, C(C*H*_3_)_3_), 0.85 (9H, C(C*H*_3_)_3_), 0.85 (d, *J* = 12 Hz, 3H, 3 × Ad*H*), 0.04 (s, 3H, Si(C*H*_3_)), −0.10 (s, 3H, Si(C*H*_3_)). ^13^C{^1^H} NMR (101 MHz, C_6_D_6_) δ 172.99 (Ar*C*), 169.56 (Ar*C*), 169.53 (Ar*C*), 169.51 (Ar*C*), 168.11 (Ar*C*), 155.48 (Ar*C*), 155.34 (Ar*C*), 150.69 (Ar*C*), 149.92 (Ar*C*), 148.78 (Ar*C*), 148.26 (Ar*C*), 130.33 (m, *o*-Ar^F^*C*), 129.97 (Ar*C*), 129.86 (q, *J*_CF_ = 31 Hz, *m*-Ar^F^*C*), 129.77 (Ar*C*), 129.52 (Ar*C*), 129.26 (Ar*C*), 126.63 (Ar*C*), 126.60 (Ar*C*), 126.54 (Ar*C*), 126.49 (Ar*C*), 126.13 (Ar*C*), 125.21 (q, *J*_CF_ = 270 Hz, *C*F_3_), 113.56 (sept, *J*_CF_ = 3 Hz, *p*-Ar^F^*C*), 112.72 (Ar*C*), 111.56 (Ar*C*), 73.48 (pz_2_-*C*H), 64.88 (Ad*C*–N), 51.33 (Ph_2_CH_3_*C*), 51.16 (Ph_2_CH_3_*C*), 36.51 (Ad*C*H_2_), 33.35 (Ph_2_*C*H_3_C), 32.42 (Ph_2_*C*H_3_C), 31.77 ((*C*H_3_)_3_C), 31.08 ((*C*H_3_)_3_C), 29.68 (Ad*C*H), 27.27 (*C*H-Co), 27.20 (Ad*C*H_2_), 20.90 ((CH_3_)_3_*C*), 20.88 ((CH_3_)_3_*C*), –1.58 (Si(*C*H_3_)), –1.90 (Si(*C*H_3_)). ^19^F NMR (282 MHz, C_6_D_6_): δ −63.38 (s, *C*F_3_). Signals for 25 of the expected 26 Ar*C* atoms are observed; one is presumably obscured due to accidental equivalence or by C_6_D_6_. FTIR: cm^–1^ 3053w, 3018w, 2971m, 2924m, 2899m, 2846m, 1594m, 1521w, 1490m, 1444m, 1406w, 1358s, 1297m, 1271s, 1237m, 1162s, 1119s, 1096m, 1074s, 1044s, 1026s, 995w, 939w, 915m, 894m, 843m, 811m, 769m, 748m, 724m, 695m, 680s, 664m, 652m, 625m, 595w, 548m, 520m, 509m, 494m, 439m. UV–vis (C_6_H_6_): λ_max_ (nm) ε_max_ (M^–1^ cm^–1^) 408 (4.82 × 10^3^), 590 (290), 754 (500), 940 sh.

## Supporting Information Available

Spectroscopic data, additional figures, and coordinates for all calculated structures (PDF)

## Supplementary Material

om3c00473_si_001.pdf

om3c00473_si_002.xyz
