# Supplementary material for: Four-Coordinate Co(III) Imide with an Unusually Tilted Terminal Imido Ligand
Source: Organometallics. 2024 Jan 12;43(3):341–8. doi: 10.1021/acs.organomet.3c00473 (PMC10865434; doi:10.1021/acs.organomet.3c00473)
Supplement: Supplementary file 1 — om3c00473_si_001.pdf [file om3c00473_si_001.pdf]

Supporting Information for:

A Four-coordinate Co(III) Imide with an Unusually Tilted Terminal Imido Ligand

*Li Gu,[1] Addison Fraker, [1] Niklas B. Thompson[2] and Alex McSkimming[1]**

[1]Department of Chemistry, Tulane University, New Orleans, LA 70118.

[2]Chemical Sciences and Engineering Division, Argonne National Laboratory, Lemont, Illinois 60439, United States

*amcskimming@tulane.edu

Contents

Spectroscopic Data S3
Additional Figures S16

Spectroscopic Data

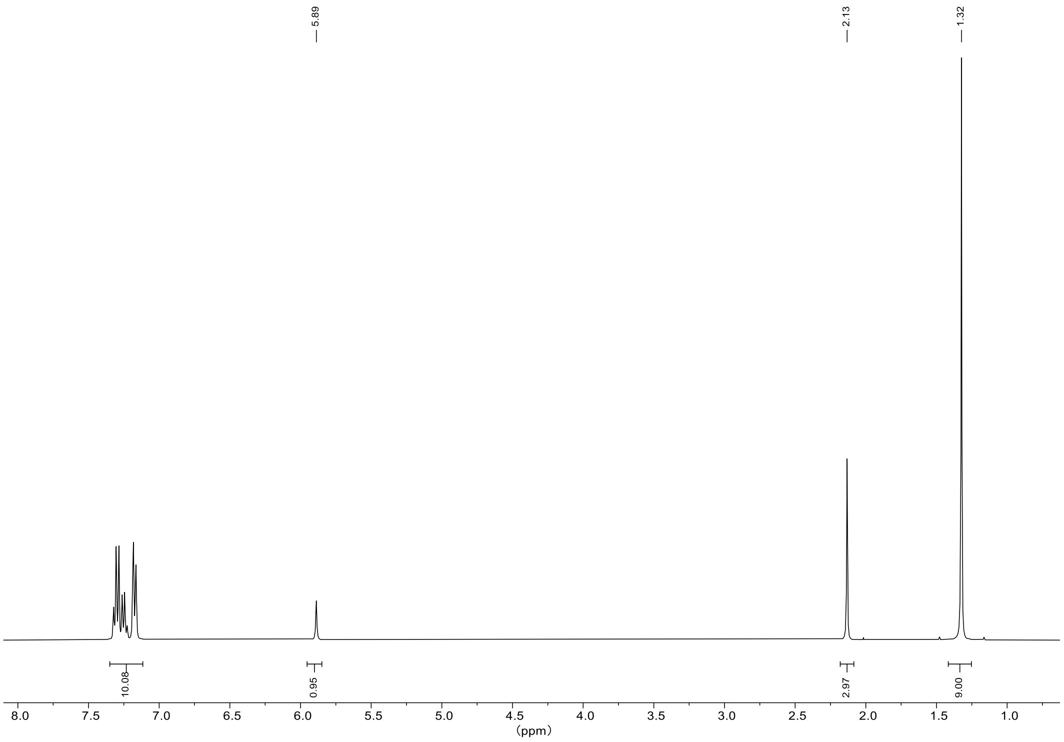

Figure S1. ^{1}H NMR spectrum of 3-tBu-5-$Ph_2(CH_3)C$-pyrazole recorded in $CDCl_3$ at 300 MHz.

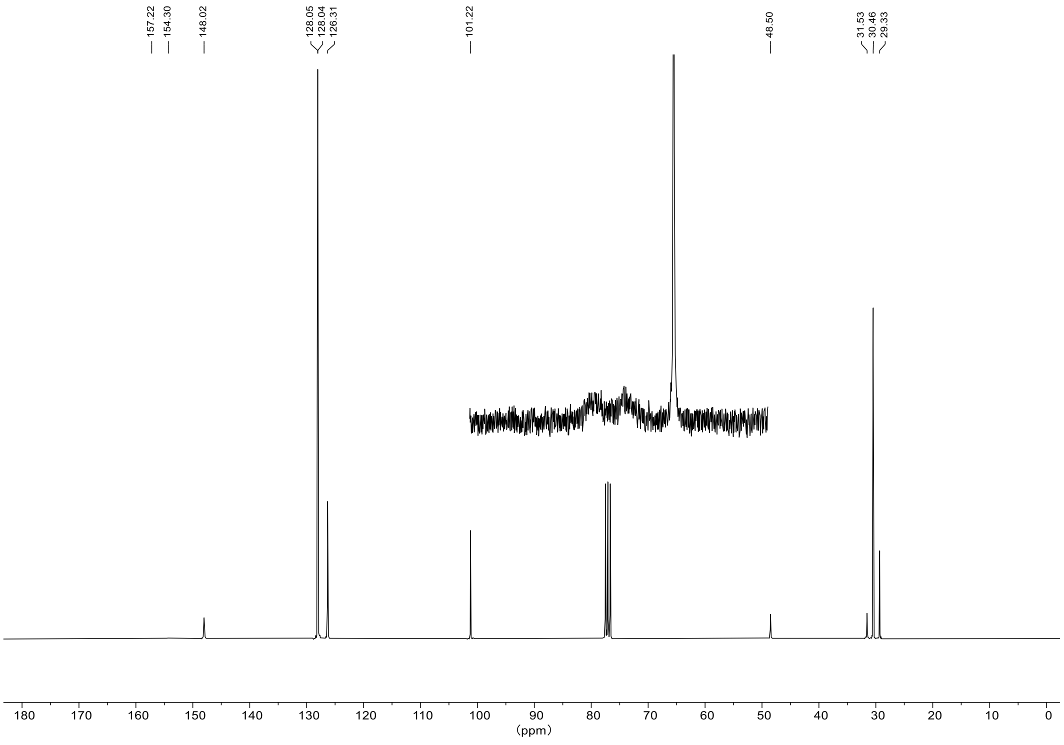

Figure S2. $^{13}C\{^{1}H\}$ NMR spectrum of 3-tBu-5-$Ph_2(CH_3)C$-pyrazole recorded in $CDCl_3$ at 101 MHz. Downfield region inset.

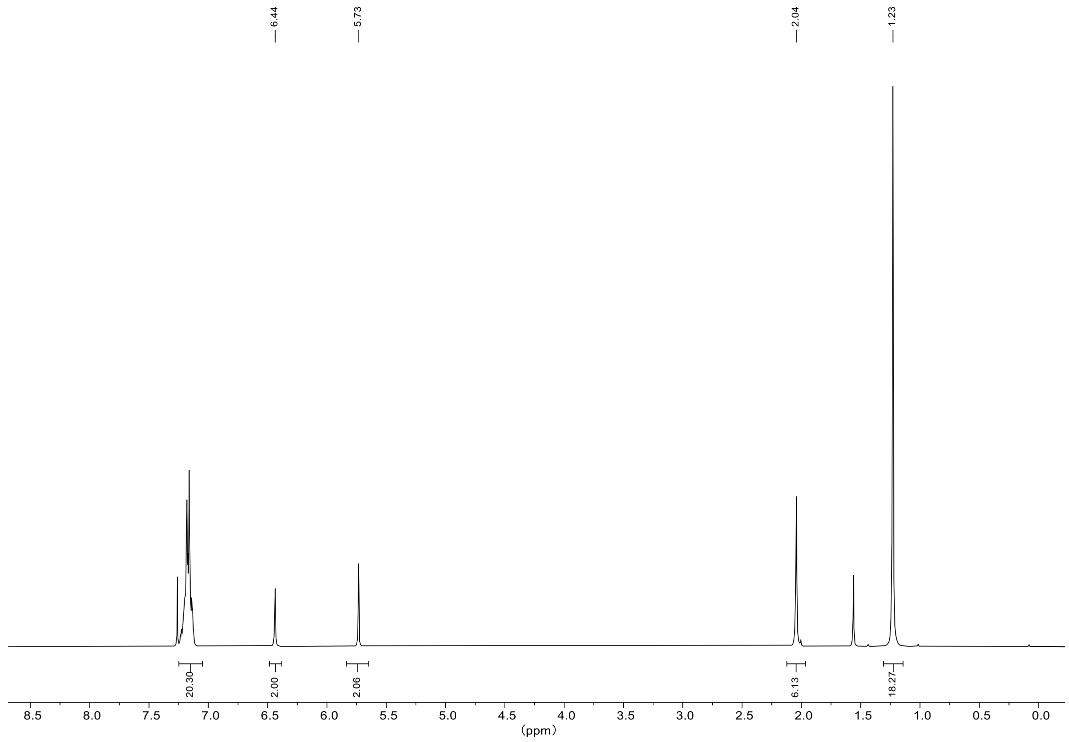

Figure S3. ^{1}H NMR spectrum of (5-tBu-3-$Ph_2(CH_3)C$-pyrazole)$_2CH_2$ recorded in $CDCl_3$ at 300 MHz.

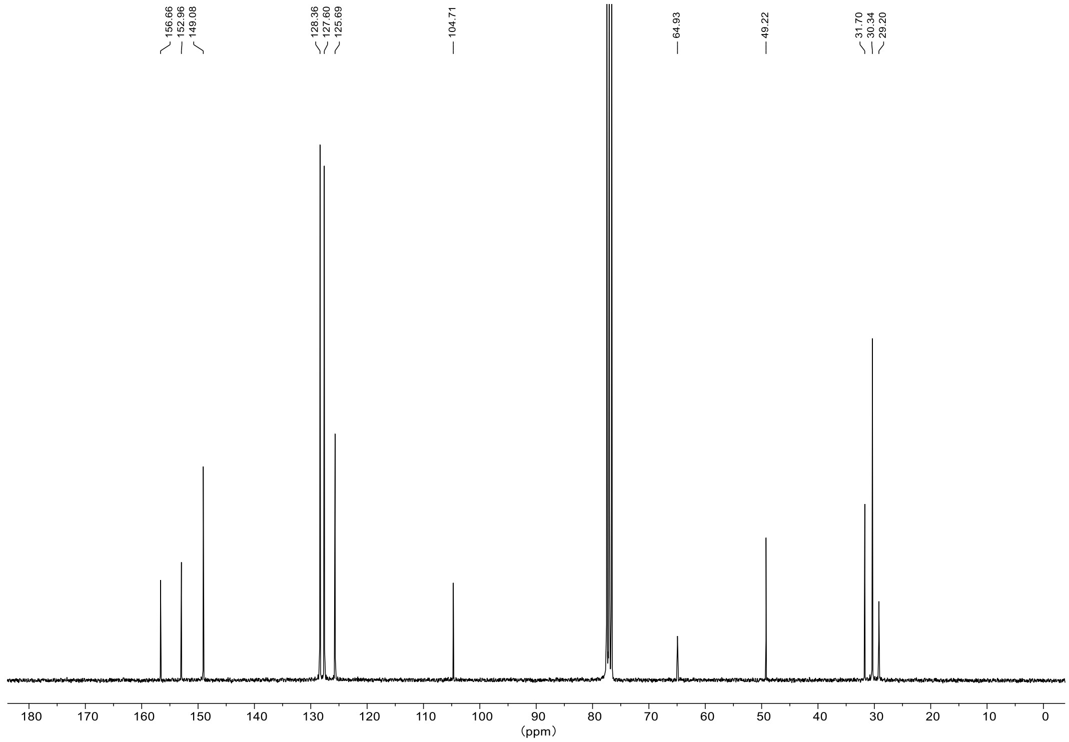

Figure S4. $^{13}C\{^{1}H\}$ NMR spectrum of (5-tBu-3-$Ph_2(CH_3)C$-pyrazole)$_2CH_2$ recorded in $CDCl_3$ at 101 MHz.

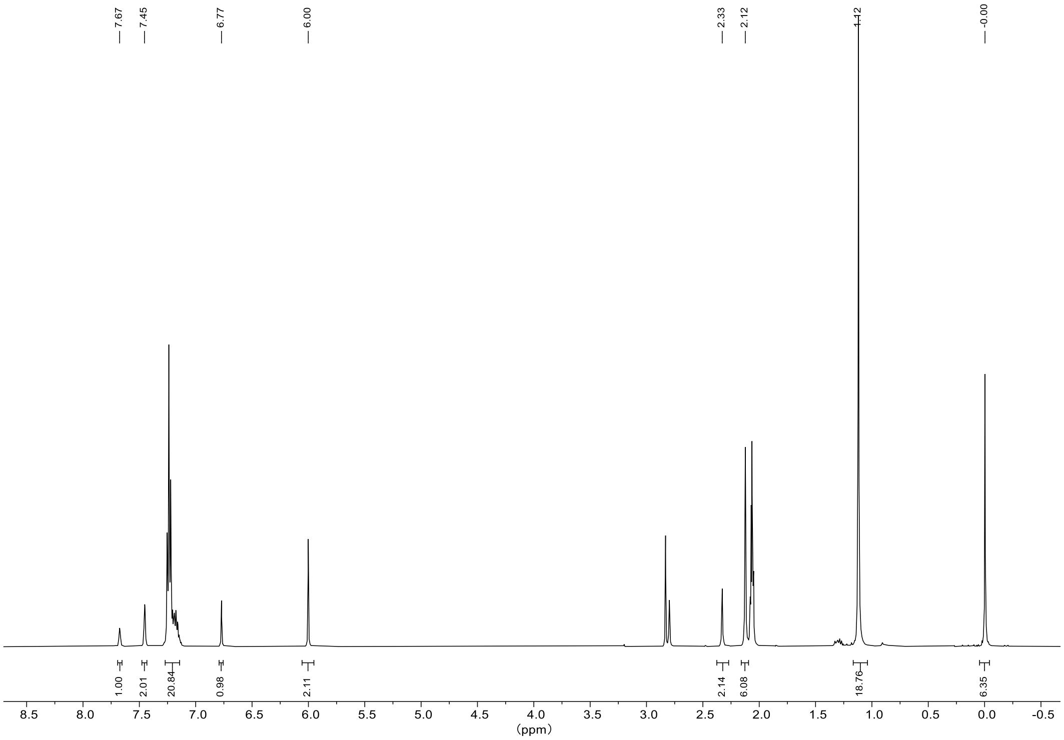

Figure S5. ^{1}H NMR spectrum of ^{dpe}**L**H recorded in $(CD_3)_2CO$ at 300 MHz.

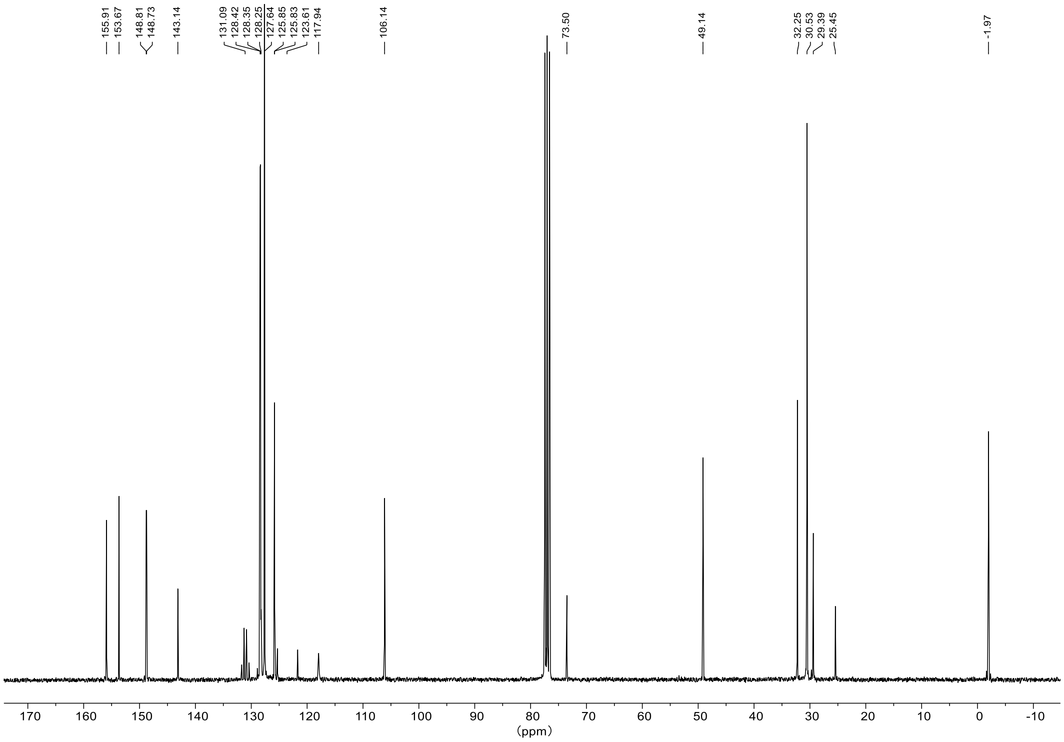

Figure S6. ^{13}C{^{1}H} NMR spectrum of ^{dpe}**L**H recorded in $CDCl_3$ at 101 MHz.

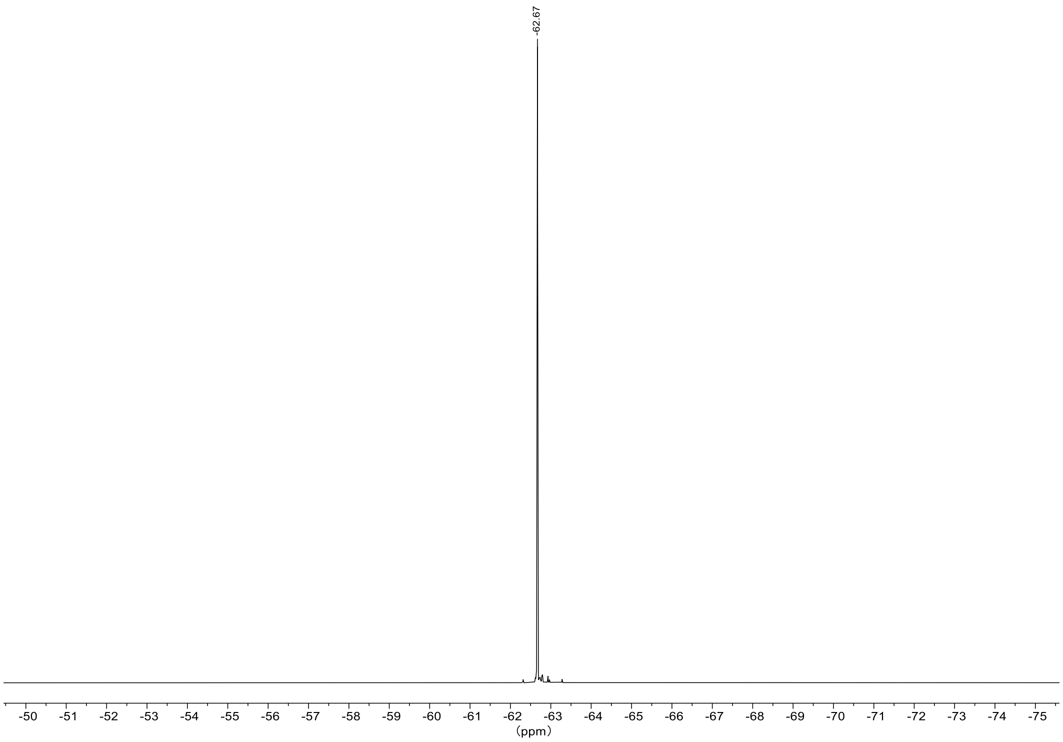

Figure S7. ^{19}F NMR spectrum of ^{dpe}**L**H recorded in $(CD_3)_2CO$ at 282 MHz.

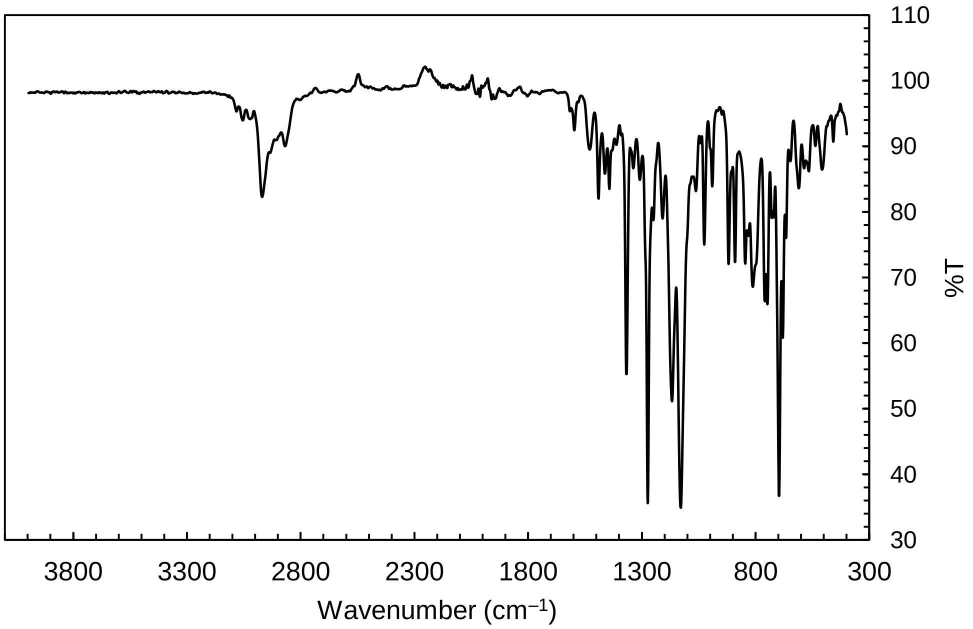

Figure S8. FTIR spectrum of ^{dpe}**L**H.

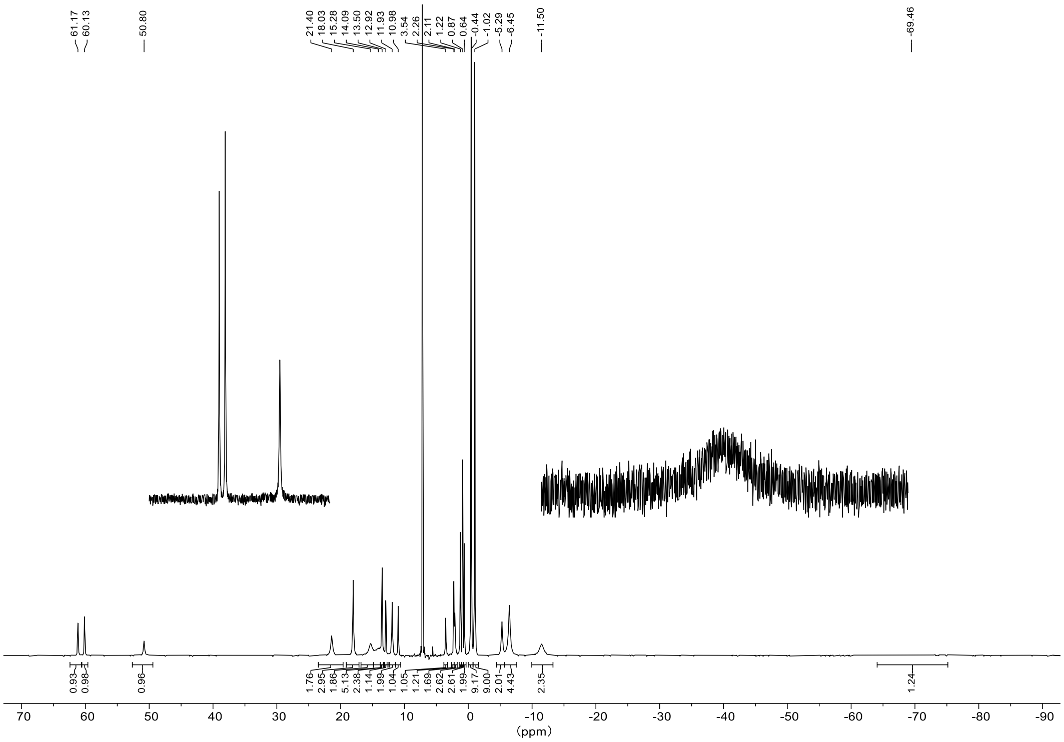

Figure S9. ^{1}H NMR spectrum of (^{dpe}**L**)CoI recorded in C_6D_6 at 300 MHz. Upfield and downfield regions inset.

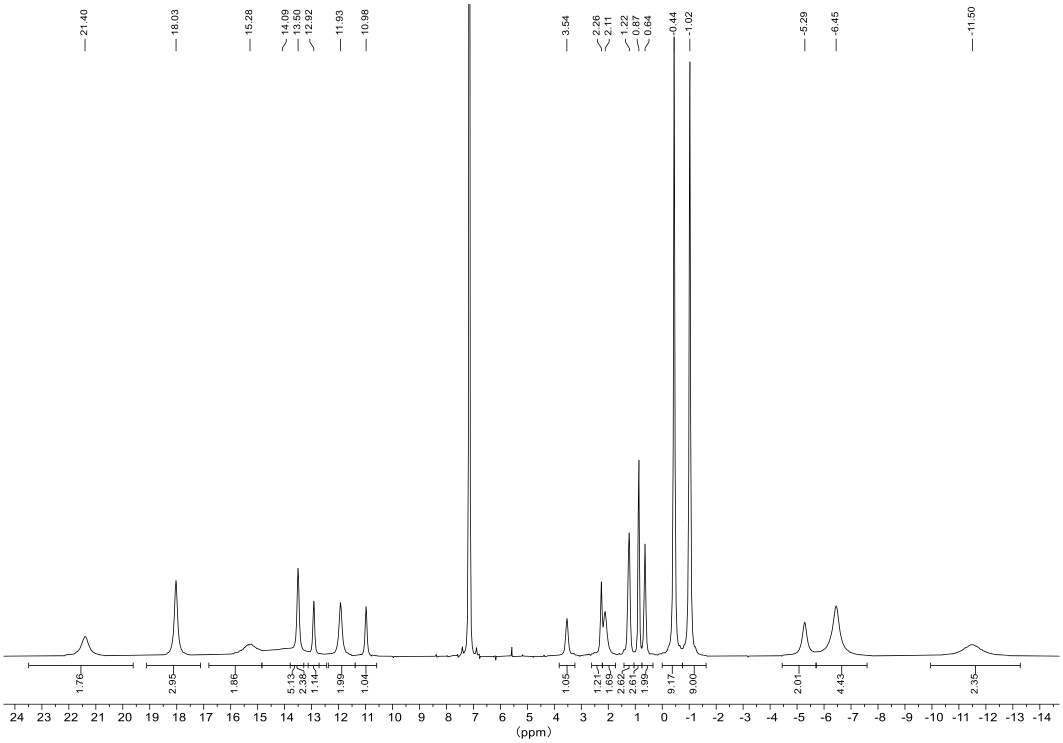

Figure S10. ^{1}H NMR spectrum of (^{dpe}**L**)CoI recorded in C_6D_6 at 300 MHz, middle region.

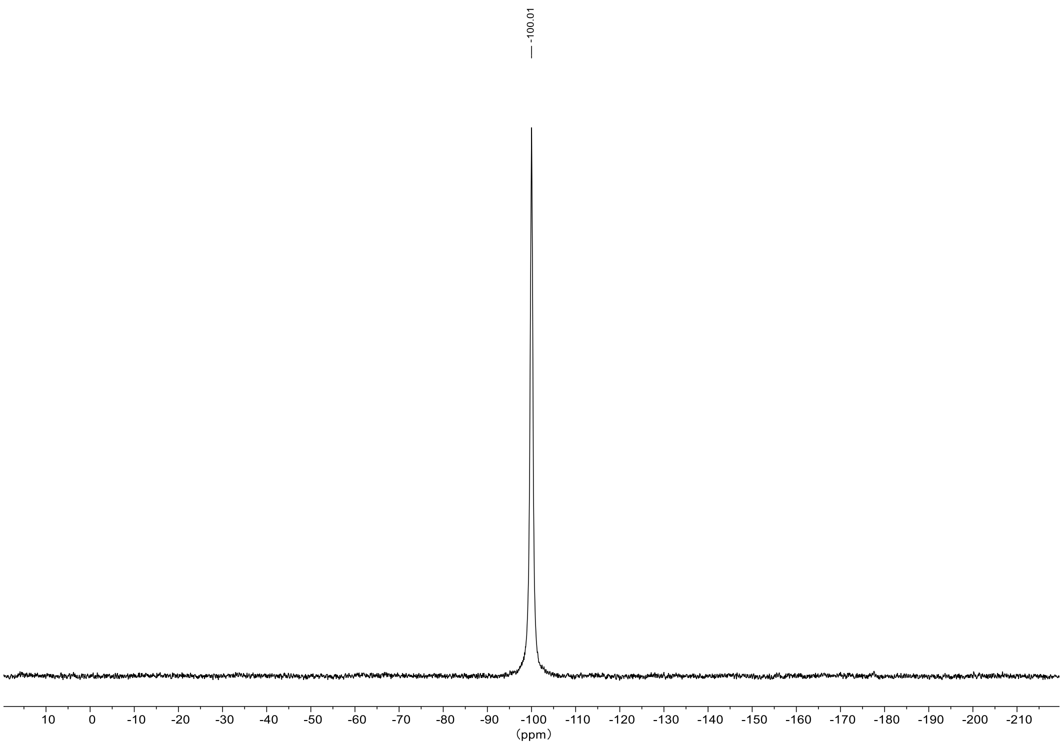

Figure S11. ^{19}F NMR spectrum of (^{dpe}**L**)CoI recorded in C_6D_6 at 282 MHz.

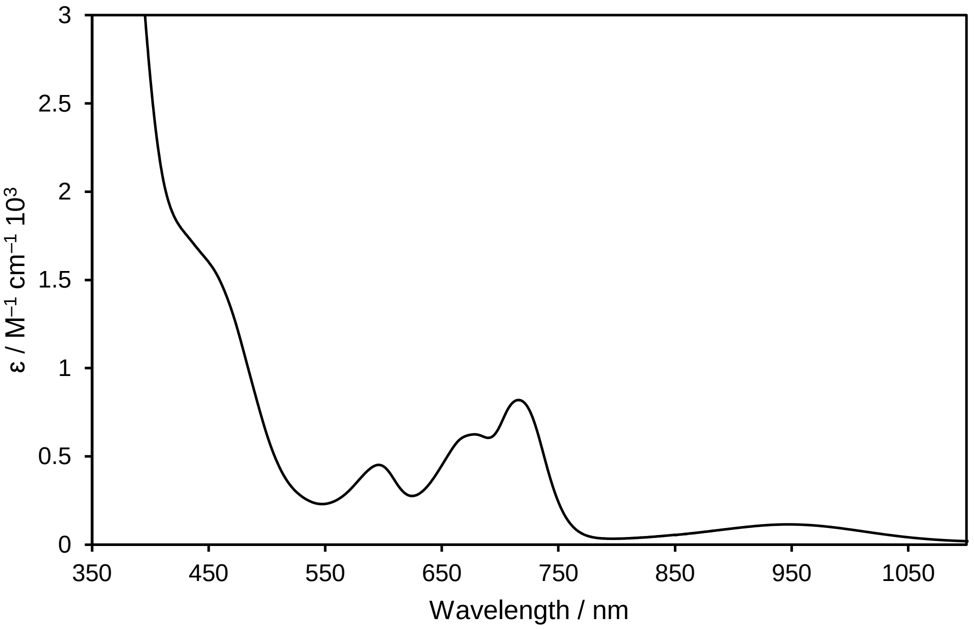

Figure S12. UV-Vis spectrum of (^{dpe}**L**)CoI in C_6H_6.

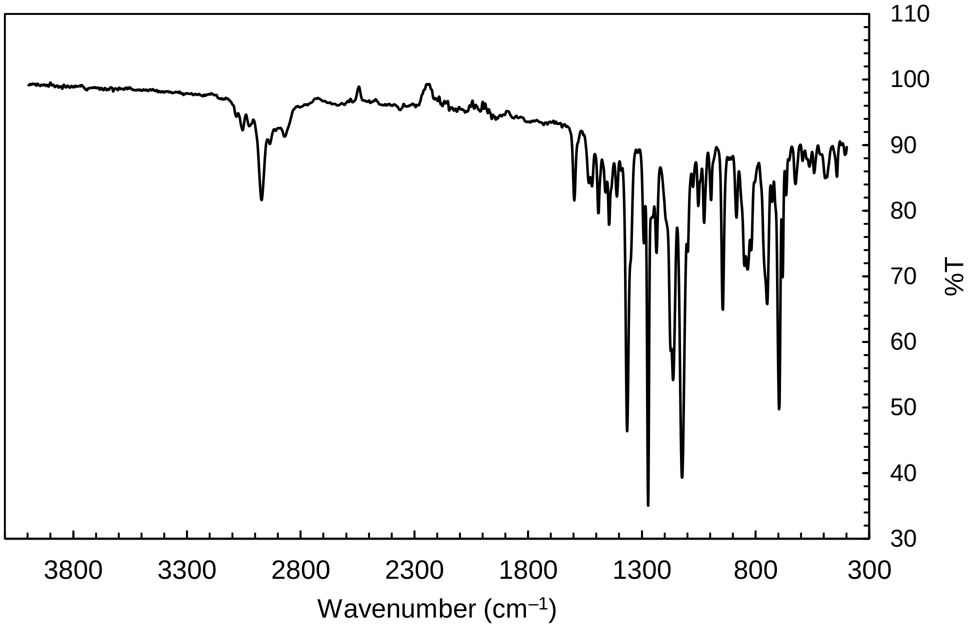

Figure S13. FTIR spectrum of (^{dpe}**L**)CoI.

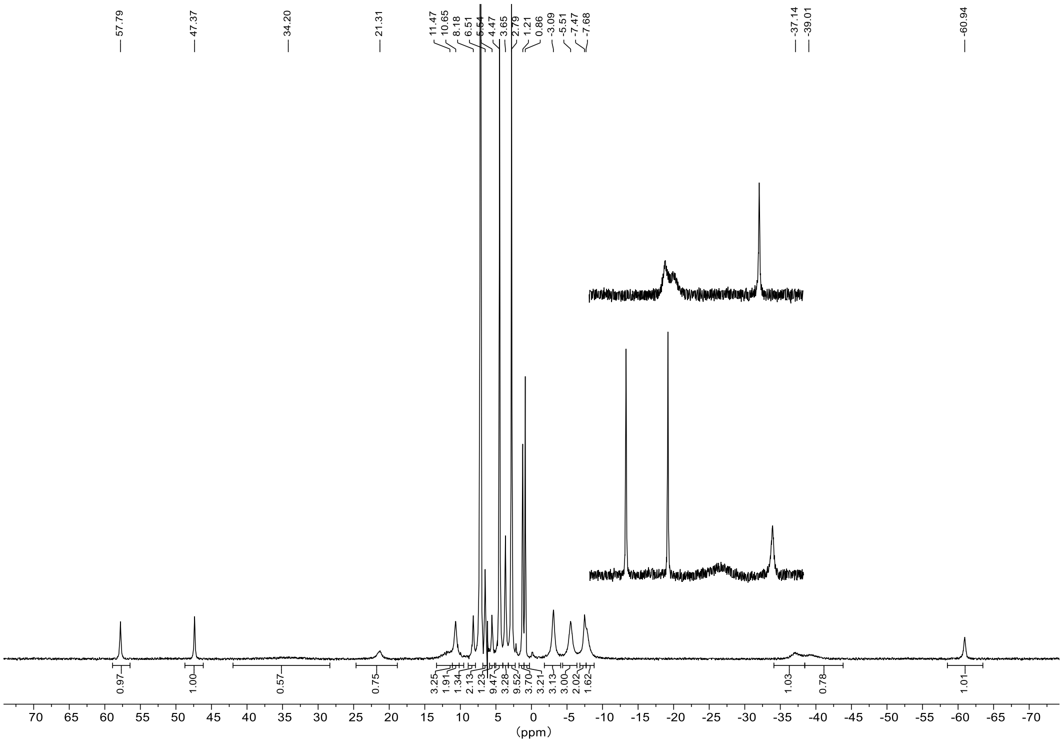

Figure S14. ^{1}H NMR spectrum of (^{dpe}**L**)Co recorded in C_6D_6 at 300 MHz. Upfield and downfield regions inset.

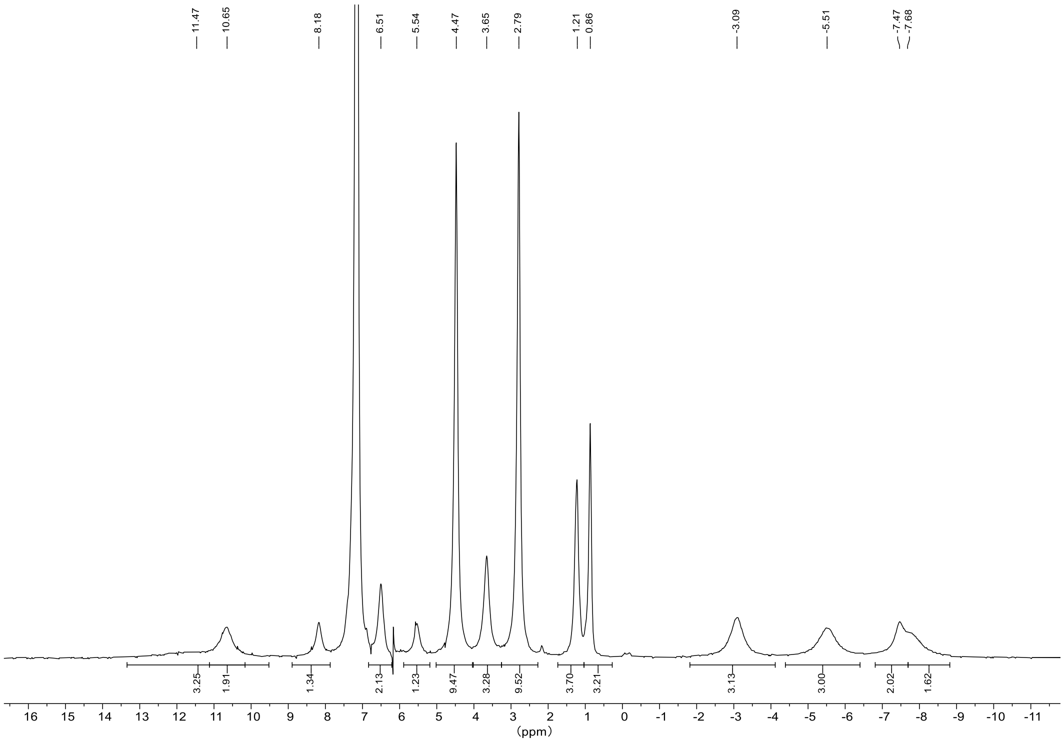

Figure S15. ^{1}H NMR spectrum of (^{dpe}**L**)Co recorded in C_6D_6 at 300 MHz, middle region.

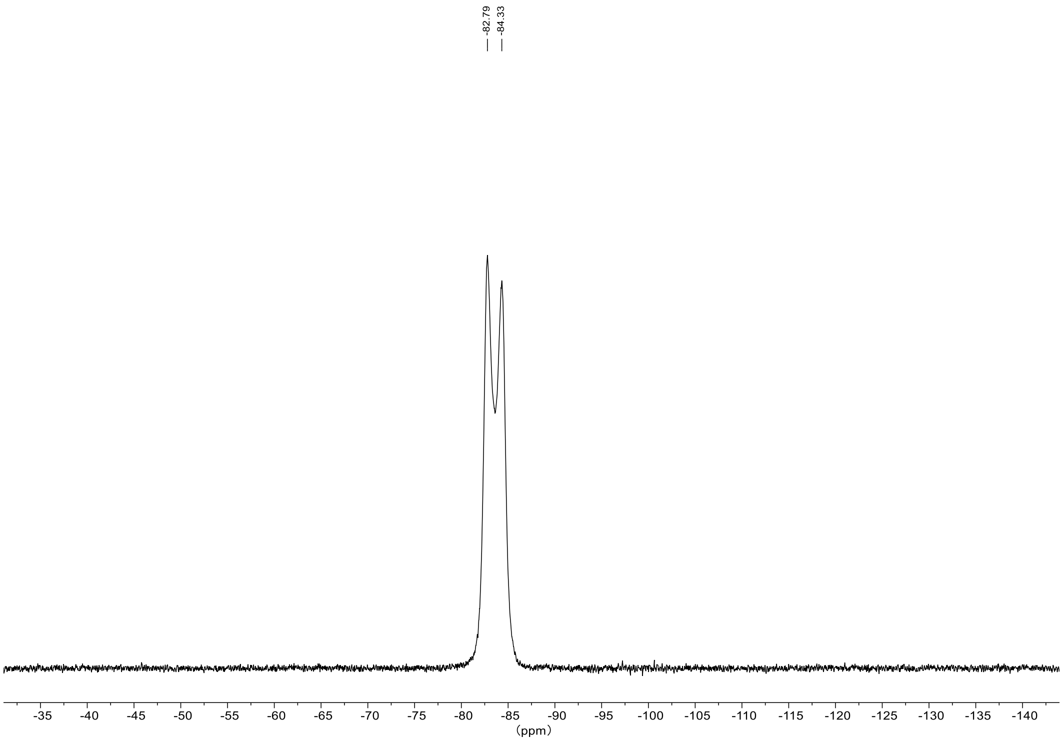

Figure S16. ^{19}F NMR spectrum of (^{dpe}**L**)Co recorded in C_6D_6 at 282 MHz.

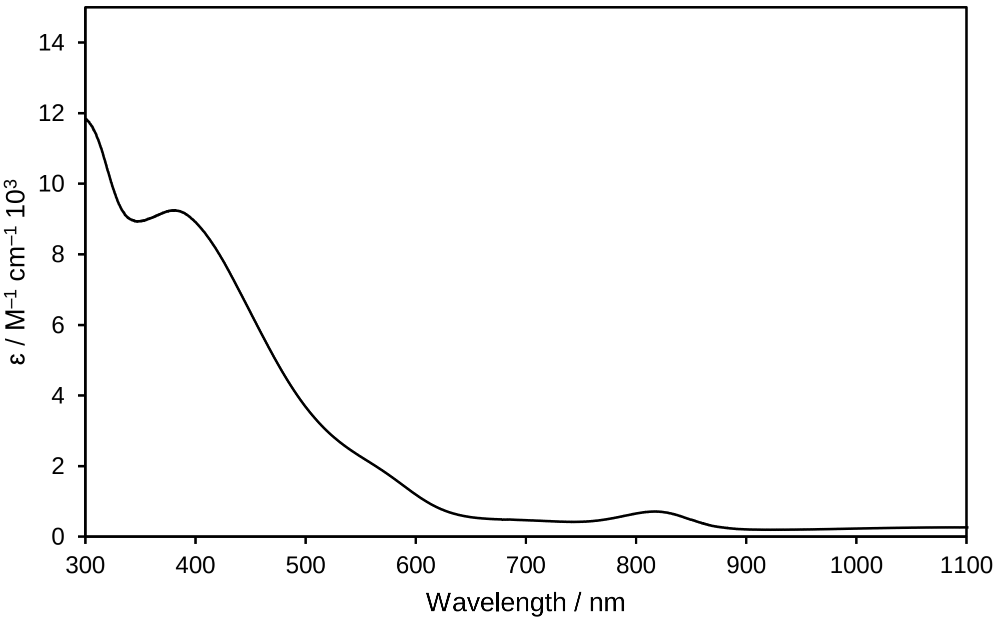

Figure S17. UV-Vis spectrum of (^{dpe}**L**)Co in C_6H_6.

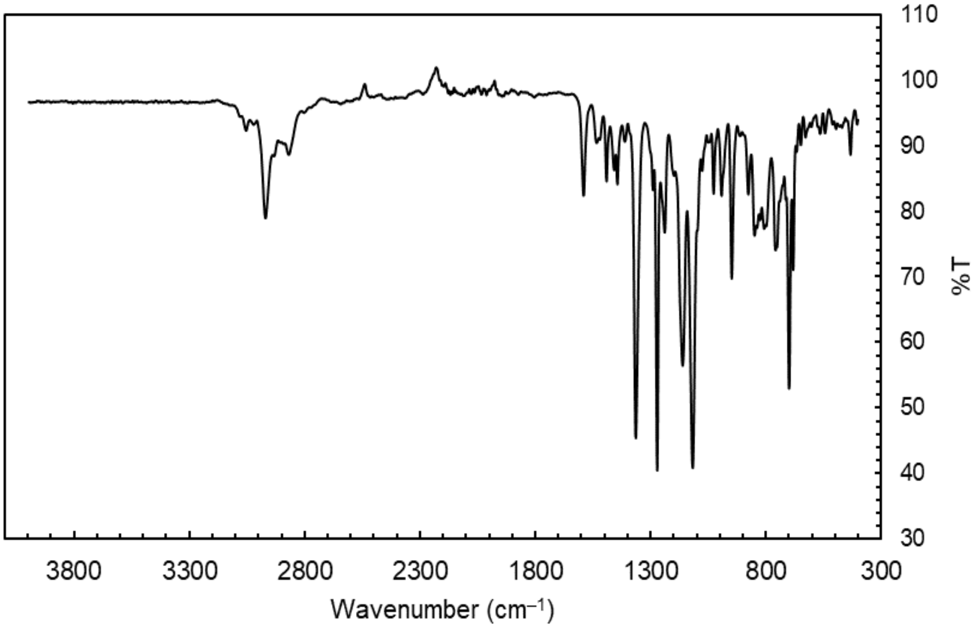

Figure S18. FTIR spectrum of (^{dpe}**L**)Co.

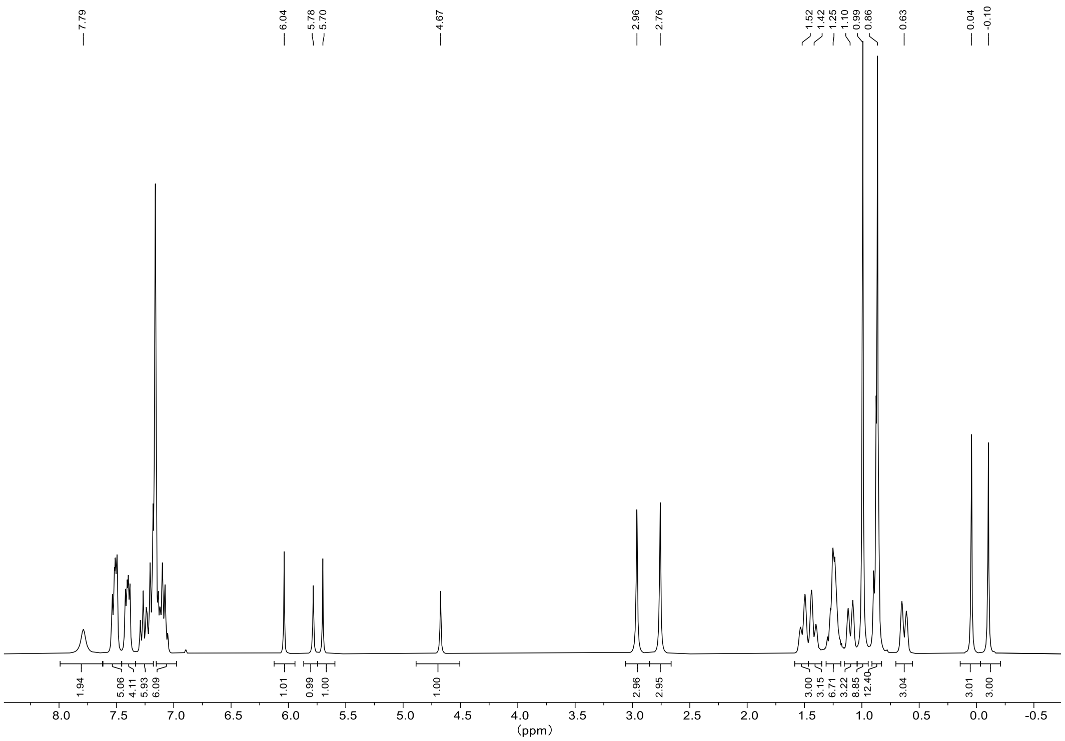

Figure S19. ^{1}H NMR spectrum of (^{dpe}**L**)CoNAd recorded in C_6D_6 at 300 MHz.

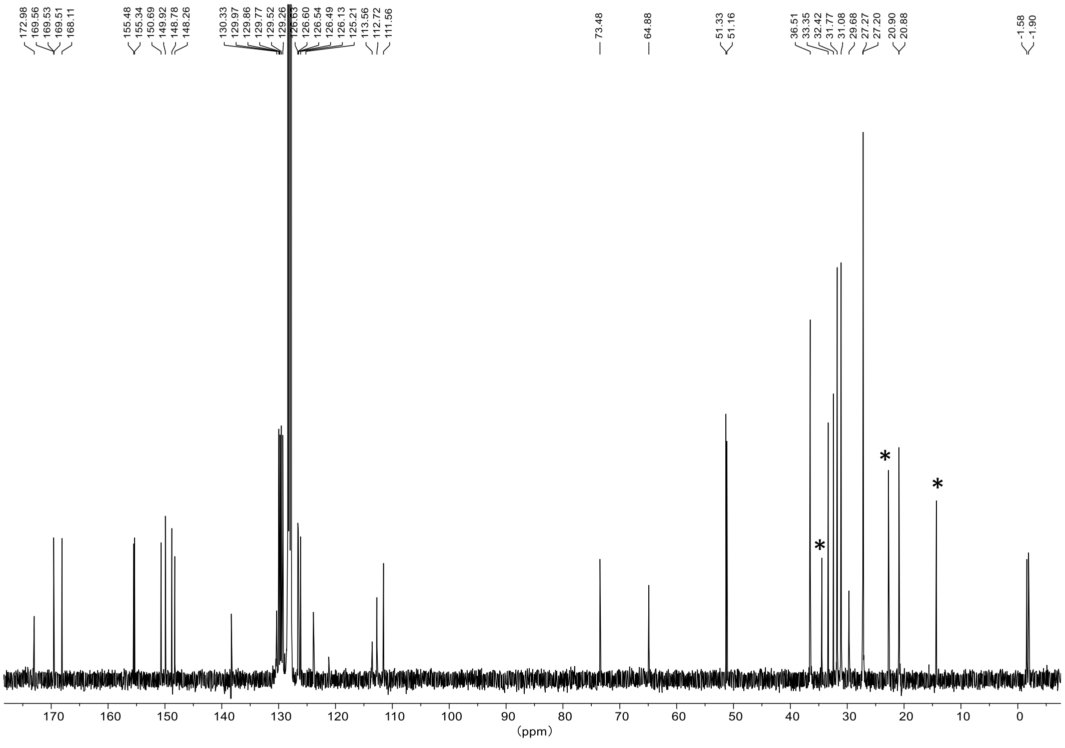

Figure S20. ^{13}C{^{1}H} NMR spectrum of (^{dpe}**L**)CoNAd recorded in C_6D_6 at 101 MHz. * Mark n-pentane of crystallization.

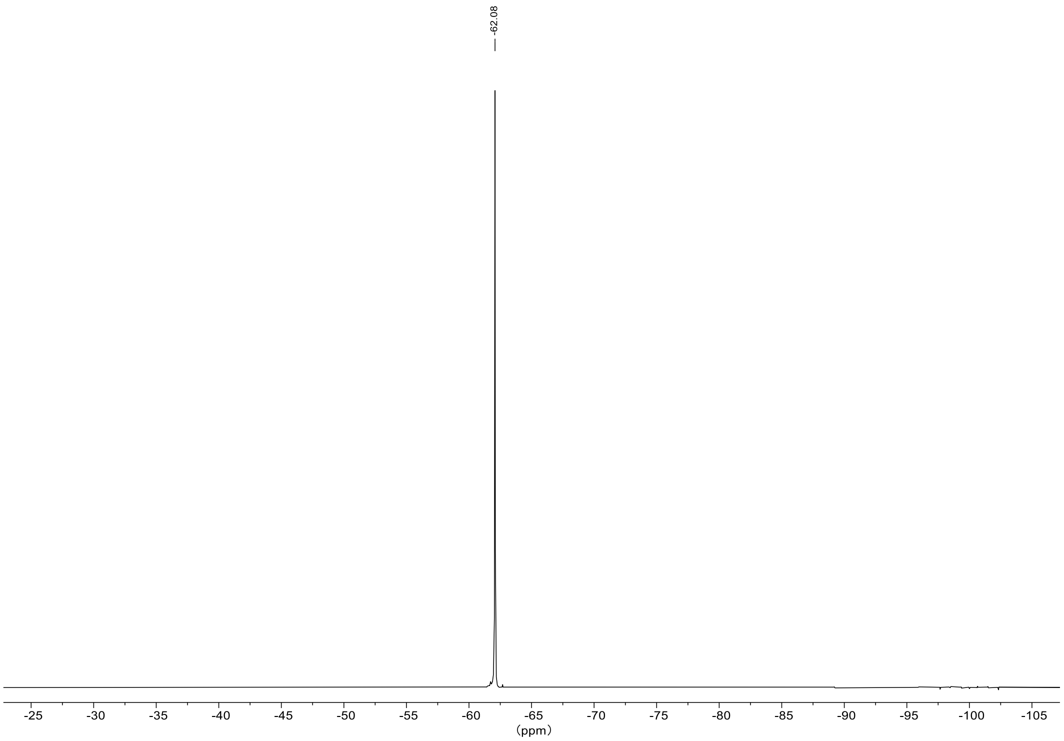

Figure S21. ^{19}F NMR spectrum of (^{dpe}**L**)CoNAd recorded in C_6D_6 at 282 MHz.

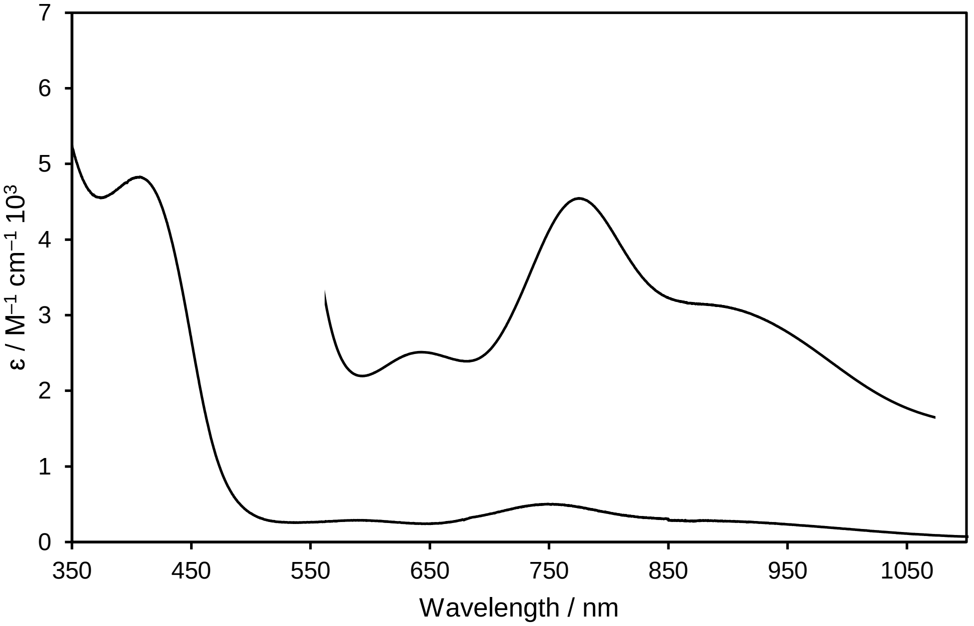

Figure S22. UV-Vis spectrum of (^{dpe}**L**)CoNAd in C_6H_6. Closeup, inset.

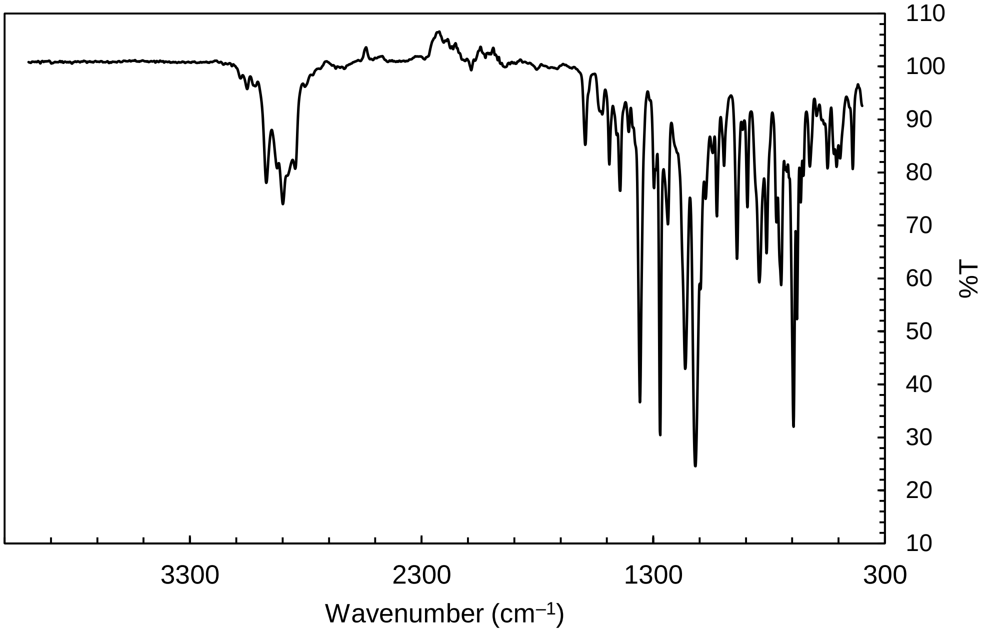

Figure S23. FTIR spectrum of (^{dpe}**L**)CoNAd.

Additional Figures

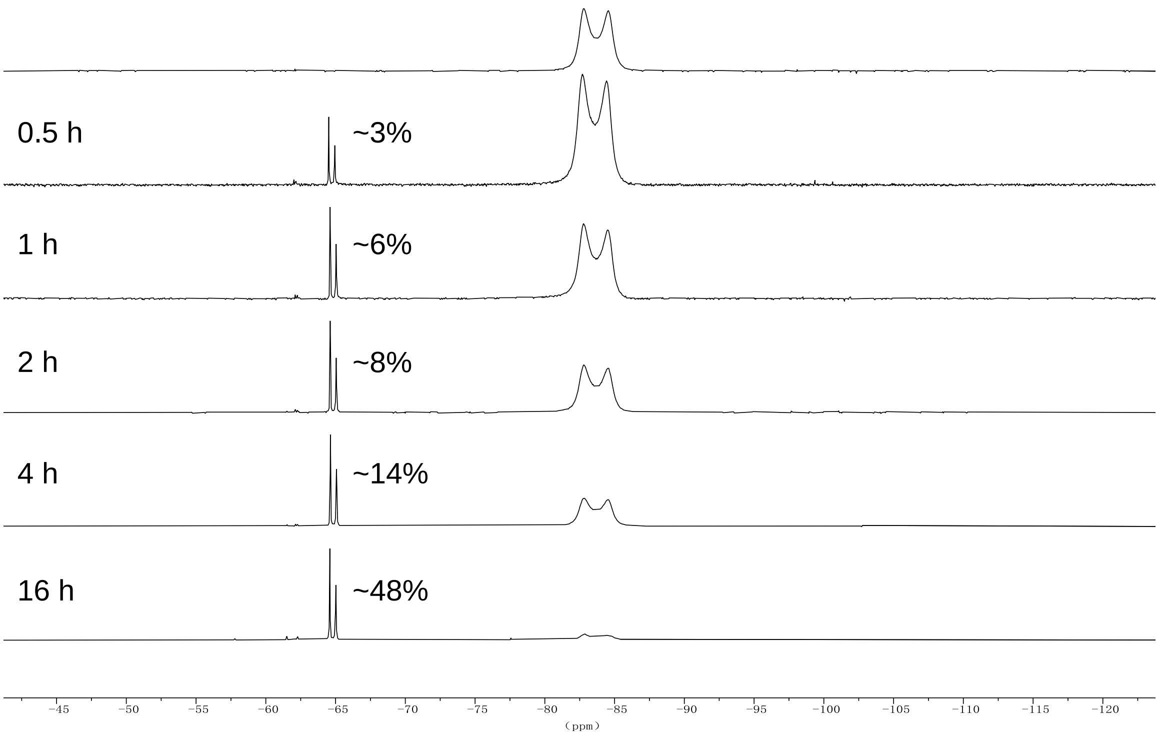

Figure S24. Thermal stability of (^{dpe}**L**)Co at 50 °C in C_6H_6. % values were determined for the combined diamagnetic impurities by integration. No paramagnetic impurities were detected.

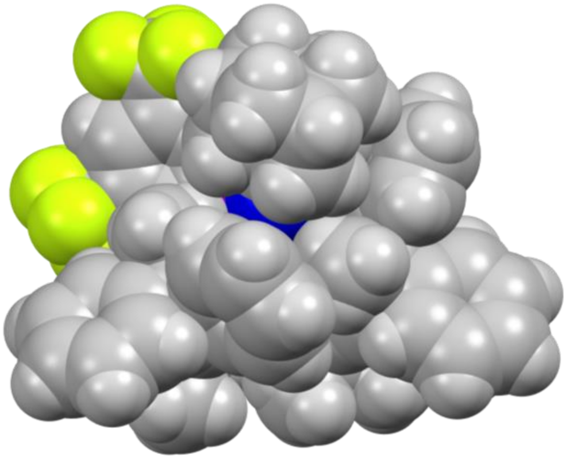

Figure S25. Space-filling model for (^{dpe}**L**)CoNAd showing the degree of ensconcement of the imido ligand.

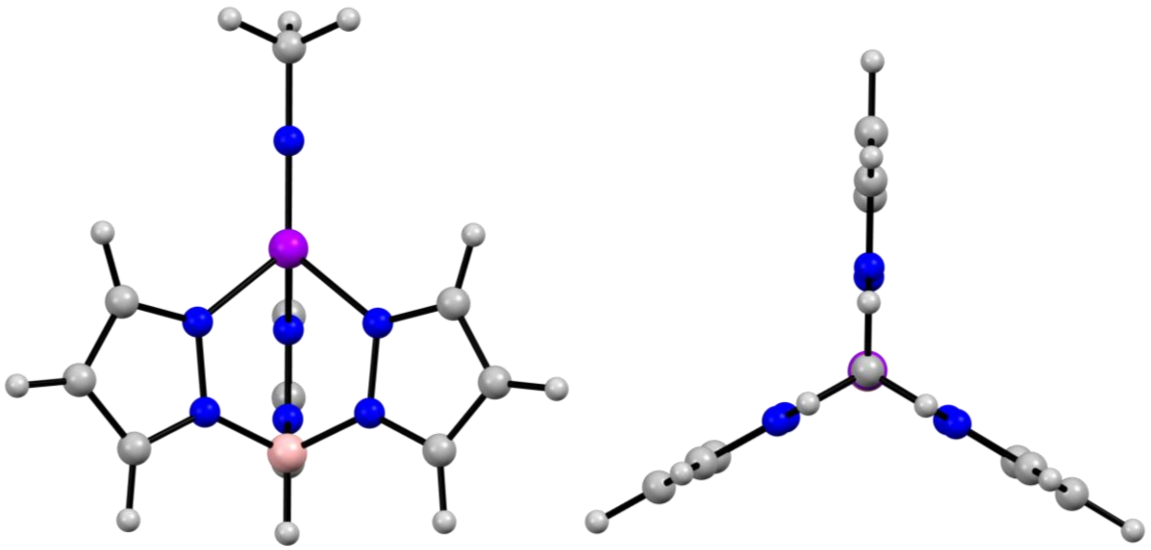

Figure S26. Calculated structures for (Tp)CoNCH$_3$ emphasizing the three-fold symmetry.

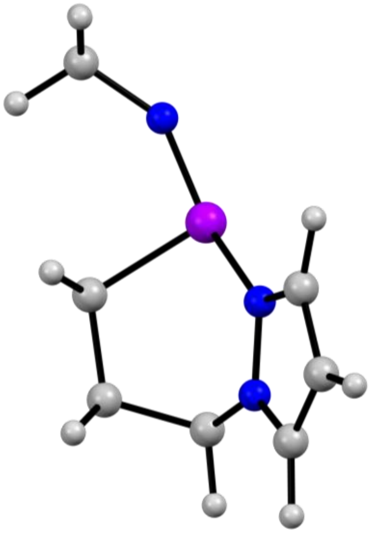

Figure S27. Calculated structure for (***L**)CoNCH$_3$ with Si replaced by C. A side-on view is presented to emphasize the acute C$_{alkyl}$–Co–N$_{im}$ angle of 99°.

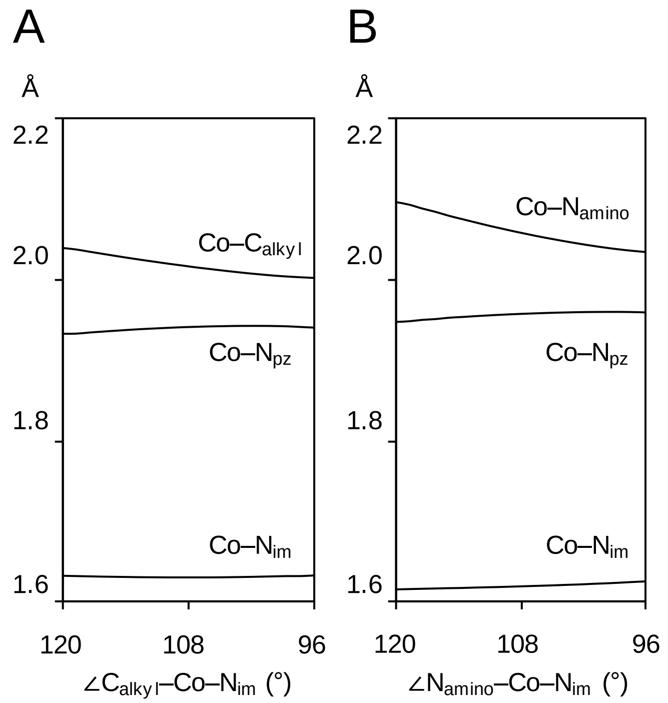

Figure S28. Trends in bond lengths for A) (***L**)CoNCH$_3$ and B) (****L**)CoNCH$_3$ as the imido ligand tilts towards the A) C$_{alkyl}$ or B) N$_{amino}$ donor.

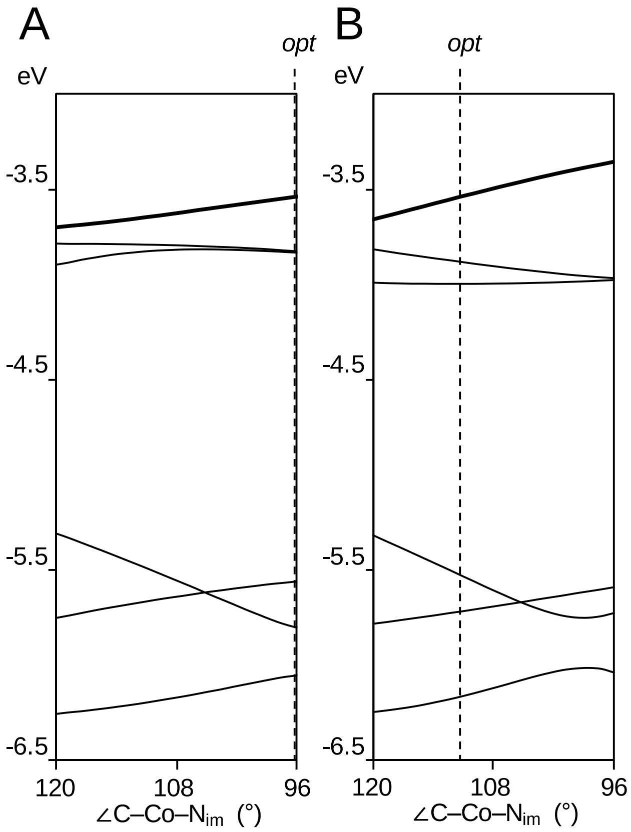

Figure S29. Walsh diagrams for (***L**)$CoNCH_3$ as the imido ligand tilts towards the C_{alkyl} donor. A) Unrestricted calculation, reproduced from the main text for clarity; B) constraining the Co–N_{im}–CH_3 angle to be 180°. The identities of the orbitals are the same as discussed in the main text. The HOMO has been highlighted for emphasis.
